# The KCNE Tango – How KCNE1 Interacts with Kv7.1

**DOI:** 10.3389/fphar.2012.00142

**Published:** 2012-08-02

**Authors:** Eva Wrobel, Daniel Tapken, Guiscard Seebohm

**Affiliations:** ^1^Cation Channel Group, Department of Biochemistry I, Faculty of Chemistry and Biochemistry, Ruhr University BochumBochum, Germany; ^2^Myozelluläre Elektrophysiologie und Molekularbiologie, Westfälische Wilhelms-Universität MünsterMünster, Germany; ^3^Department of Drug Design and Pharmacology, University of CopenhagenCopenhagen, Denmark

**Keywords:** Kv channel, Kv7.1, β-subunit, KCNE1

## Abstract

The classical tango is a dance characterized by a 2/4 or 4/4 rhythm in which the partners dance in a coordinated way, allowing dynamic contact. There is a surprising similarity between the tango and how KCNE β-subunits “dance” to the fast rhythm of the cell with their partners from the Kv channel family. The five KCNE β-subunits interact with several members of the Kv channels, thereby modifying channel gating via the interaction of their single transmembrane-spanning segment, the extracellular amino terminus, and/or the intracellular carboxy terminus with the Kv α-subunit. Best studied is the molecular basis of interactions between KCNE1 and Kv7.1, which, together, supposedly form the native cardiac *I*_Ks_ channel. Here we review the current knowledge about functional and molecular interactions of KCNE1 with Kv7.1 and try to summarize and interpret the tango of the KCNEs.

## Introduction

Voltage-gated potassium channels (Kv) are ubiquitously expressed in human tissues. They enable the rapid, selective movement of potassium ions through cellular membranes, thereby regulating physiological processes such as transmembrane ion passage and hormone secretion, vesicle cycling, and cell excitability. Kv channels display a huge diversity due to the large number of different α-subunits, alternative splicing, post-transcriptional modifications, and their ability to form heteromeric channels with other pore-forming α-subunits. To complicate the situation even more, the number of functionally different Kv channels in native tissues is further increased by interaction with regulatory β-subunits. These accessory subunits modify subcellular localization as well as biophysical properties of Kv channels such as gating kinetics, ion selectivity, and pharmacology. Among the best studied modulations of Kv channels by regulatory β-subunits is the effect of KCNE1 on Kv7.1.

## Kv7.1-Expression and Physiological Function

**Figure 1:**
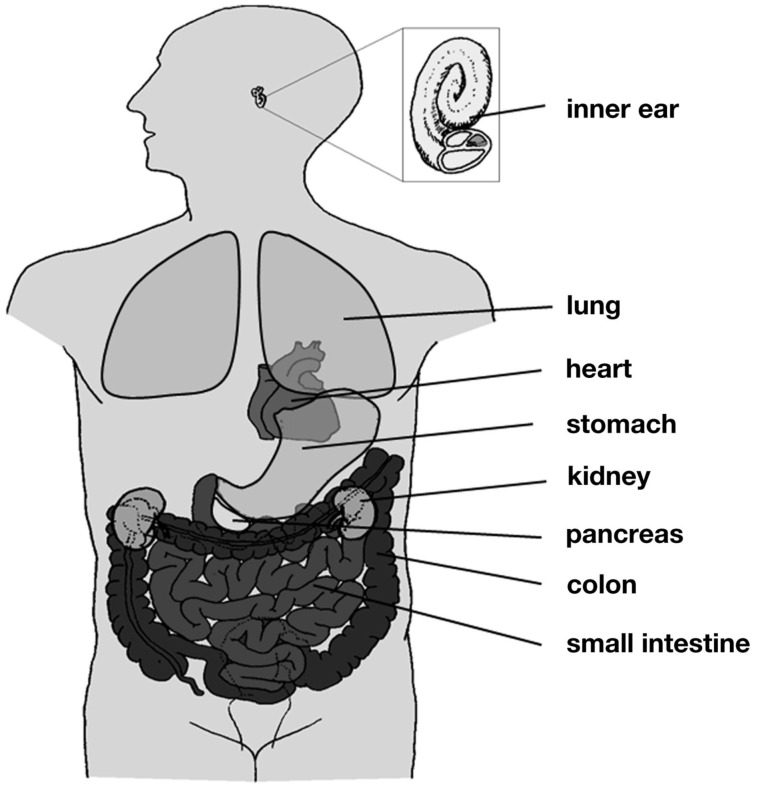
**Distribution of Kv7.1**. Kv7.1 is expressed in several tissues throughout the human body, including heart, lung, inner ear, kidney, and the gastrointestinal tract.

The *KCNQ1* gene was first identified by Wang et al. (1996b) in a linkage study of patients with long QT syndrome (LQTS1). Its gene product, Kv7.1 (also termed KvLQT1 or KCNQ1), is a voltage-gated potassium channel α-subunit, and its expression was detected in several mammalian tissues, including heart, epithelia, and smooth muscle (Figure 1; Table A1 in Appendix). Kv7.1 can assemble with different members of the KCNE family of regulatory β-subunits to fulfill a variety of physiological functions.

In the heart, Kv7.1 is involved in the termination of the cardiac action potential. The repolarizing potassium current, *I*_K_, consists of two major components, the rapid delayed rectifier potassium current *I*_Kr_ and the slow delayed rectifier potassium current *I*_Ks_ (Sanguinetti and Jurkiewicz, 1990). Kv7.1 coassembles with the KCNE1 β-subunit to form the channel complex that mediates *I*_Ks_ (Barhanin et al., 1996; Sanguinetti et al., 1996). Although KCNE1 is the major accessory subunit assembling with Kv7.1 in the heart, other subunits of the KCNE family might be present (Bendahhou et al., 2005), serving as additional regulators of *I*_Ks_ (Wu et al., 2006). The significance of Kv7.1 and its accessory β-subunits for maintaining normal rhythmicity is further emphasized by the numerous *KCNQ1* and *KCNE* mutations associated with cardiac arrhythmias (http://www.fsm.it/cardmoc/). Most of these mutations lead to loss of channel function causing LQTS, a disorder predisposing affected individuals to *torsade de pointes* arrhythmia and cardiac sudden death.

Besides its cardiac function, several lines of evidence suggest an important role of Kv7.1 and its accessory β-subunit KCNE1 in the hearing process. In patients suffering from Jervell and Lange-Nielsen syndrome – the recessive form of inherited LQTS – cardiac arrhythmia is accompanied by profound bilateral deafness. Mutations in both *KCNQ1* and *KCNE1* genes have been reported to cause this disorder (Jervell and Lange-Nielsen, 1957; Neyroud et al., 1997; Schulze-Bahr et al., 1997). In addition, targeted disruption of the *KCNQ1* gene in mice leads to deafness caused by morphological abnormalities of the inner ear (Lee et al., 2000; Casimiro et al., 2001). Expression of Kv7.1 and KCNE1 has been detected in the marginal cells of the *stria vascularis* of the cochlea and the vestibular dark cells (Neyroud et al., 1997; Nicolas et al., 2001; Knipper et al., 2006; Hur et al., 2007). Both cell types are involved in the generation of the potassium-rich endolymph, and Kv7.1/KCNE1 channels have been suggested to be key mediators of this K^+^ secretion (Marcus and Shen, 1994; Shen et al., 1995; Wangemann, 1995; Wangemann et al., 1995; Sunose et al., 1997).

In addition to the inner ear epithelium, Kv7.1 has been detected in a variety of other epithelial cell types, where it participates in secretory transduction. In the kidney, Kv7.1/KCNE1 channels seem to be located in the proximal tubule of the nephron (Sugimoto et al., 1990; Vallon et al., 2001), conducting a K^+^ current to counterbalance membrane depolarization induced by electrogenic Na^+^-coupled transport of glucose or amino acids (Vallon et al., 2001, 2005). The relevance of Kv7.1/KCNE1 channels for renal function is further underlined by the observation that KCNE1 knockout mice suffer from hypokalemia, urinary and fecal salt wasting, and volume depletion (Arrighi et al., 2001; Warth and Barhanin, 2002). Kv7.1 expression has also been detected in the small intestine and the colon (Schroeder et al., 2000; Dedek and Waldegger, 2001; Demolombe et al., 2001; Kunzelmann et al., 2001; Horikawa et al., 2005). In colonic crypt cells Kv7.1 is believed to assemble with another accessory β-subunit, KCNE3, and to mediate a K^+^ conductance that provides the driving force for chloride secretion (Schroeder et al., 2000; Kunzelmann et al., 2001). Two further examples of Kv7.1 expression and function in chloride-secreting tissues are pancreatic acinar cells and airway epithelium (Kim and Greger, 1999; Kottgen et al., 1999; Mall et al., 2000; Demolombe et al., 2001; Grahammer et al., 2001b; Lee et al., 2004). In parietal cells of the stomach Kv7.1 coassembles with KCNE2 and participates in gastric acid secretion (Dedek and Waldegger, 2001; Demolombe et al., 2001; Grahammer et al., 2001a; Heitzmann et al., 2004). In KCNQ1 knockout mice gastric hyperplasia and profound hypochlorhydria have been observed, indicating the importance of Kv7.1 in normal stomach development and function (Lee et al., 2000). Kv7.1 expression has also been detected in the human thyroid gland, and it has been shown that mice lacking functional Kv7.1 develop hypothyroidism (Frohlich et al., 2011). Recently, Kv7.1 channels have been shown to relax systemic and pulmonary arteries upon pharmacological activation (Chadha et al., 2012).

## Regulation of Kv7.1 by Accessory β-Subunits of the KCNE Gene Family

**Figure 2:**
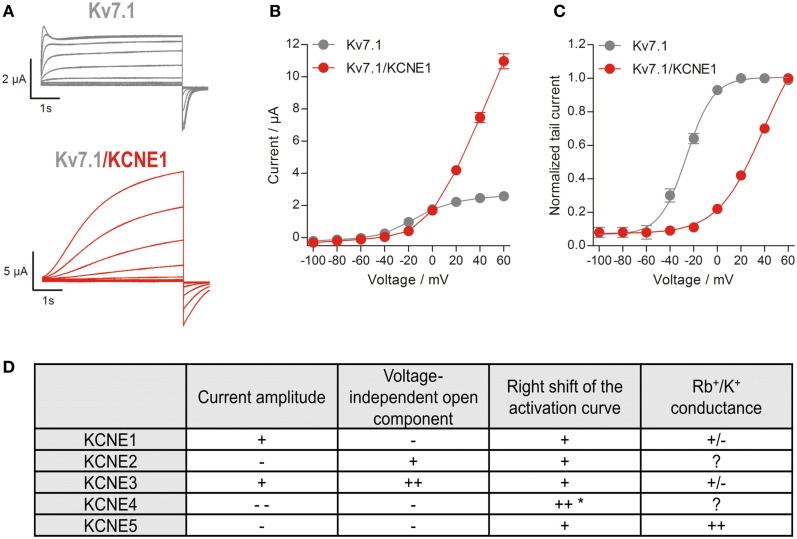
**Characteristics of Kv7.1/KCNE-mediated currents**. **(A)** Representative current traces of Kv7.1 homomers and Kv7.1/KCNE1 heteromers. Channels were expressed in *Xenopus* oocytes, and currents were elicited with 7 s pulses to potentials of −100 to +60 mV, applied in 20 mV increments from a holding potential of −80 mV. Tail currents were recorded at −120 mV. **(B)** Current-voltage relationships. At voltages between −60 and −40 mV KCNE1 suppresses currents, whereas it stimulates them at voltages above 0 mV. **(C)** Voltage dependence of channel activation determined by tail current analysis. Activation curves were fitted to a Boltzmann function. Note: Kv7.1/KCNE1 channels are not fully activated at +60 mV. **(D)** Effects of different KCNE subunits on Kv7.1 currents. “+” and “++” indicate increased and strongly increased effects, while “−” and “−−” indicate decreased and strongly decreased effects, respectively. *Effect shown in the calmodulin binding-deficient KCNE4^L69–L72^ mutant (Ciampa et al., 2011). The Rb^+^/K^+^ conductance tightly correlates with the partial inactivation, and KCNE5 slightly increases it, whereas KCNE1/3 decreases it compared to Kv7.1 (Seebohm et al., 2003c).

All five members of the KCNE family of regulatory β-subunits can functionally coassemble with Kv7.1 to subunit-specifically change its current characteristics (for an excellent review about KCNEs, see McCrossan and Abbott, 2004). The founding member KCNE1 (previously termed MinK or *I*_sK_) was identified in 1988 (Takumi et al., 1988). As its expression in *Xenopus* oocytes produced slowly activating potassium currents resembling the cardiac *I*_Ks_, KCNE1 was originally believed to be the ion channel α-subunit forming the *I*_Ks_ channel. However, it is now well established that KCNE1 cannot form functional K^+^ channels alone, but can serve as a regulatory β-subunit for several voltage-gated cation channels, including Kv7.1 (Barhanin et al., 1996; Sanguinetti et al., 1996). The *I*_Ks_-like potassium currents observed in oocytes upon heterologous expression of KCNE1 were actually caused by coassembly with endogenous Kv7.1. So far, four other members of the KCNE family, KCNE2-KCNE5 (also termed MiRP1-4) have been identified (Abbott et al., 1999; Piccini et al., 1999), and their influence on Kv7.1 channels has been studied extensively in heterologous expression systems. Kv7.1 expressed alone forms a classical delayed rectifier potassium-selective channel with fast activation, delayed partial inactivation, and relatively slow deactivation (Pusch et al., 1998; Tristani-Firouzi and Sanguinetti, 1998). The presence of KCNE1 drastically modifies Kv7.1 activity by increasing unitary conductance as well as macroscopic currents, slowing activation, right-shifting voltage dependence of activation, suppressing currents at low activating voltages, suppressing partial inactivation, increasing *Q*_10_-value, and modulating pharmacology (Figures 2A–C; Barhanin et al., 1996; Sanguinetti et al., 1996; Pusch, 1998; Pusch et al., 1998; Sesti and Goldstein, 1998; Tristani-Firouzi and Sanguinetti, 1998; Lerche et al., 2000; Seebohm et al., 2001a,b,c, 2003a,b,c, 2005; Morokuma et al., 2008). Coassembly of Kv7.1 and KCNE2 gives rise to currents with decreased amplitude, instantaneous activation, rapid partial deactivation, and a linear current-voltage relationship (Tinel et al., 2000). KCNE3 also converts Kv7.1 to a channel with nearly instantaneous activation and a linear current-voltage relationship (Schroeder et al., 2000; Seebohm et al., 2003c), but in contrast to Kv7.1/KCNE2, complexes containing KCNE3 show some time dependency of gating at positive potentials and increased current densities (Schroeder et al., 2000; Melman et al., 2001; Mazhari et al., 2002). KCNE4 completely suppresses Kv7.1 currents at physiologically relevant membrane potentials (Grunnet et al., 2002) and KCNE5 shifts the voltage dependence of Kv7.1 activation to more positive potentials toward an activation threshold of about +40 mV (Figure 2D; Angelo et al., 2002; Seebohm et al., 2003c).

## Gating of Kv7.1 and Its Modulation by KCNE1

The biophysical properties of voltage-gated Kv7.1 channels change dramatically when they coassemble with KCNEs. Homotetrameric Kv7.1 channels activate with relatively fast kinetics with τ_activation_ by about 80−100 ms at 60 mV (Figure 2; Pusch et al., 1998; Tristani-Firouzi and Sanguinetti, 1998). The channels undergo partial inactivation, with about 60% of the channels being inactivated at 60 mV in steady-state (Figure 2). The kinetic behavior of Kv7.1 can be approximated by a linear gating scheme of the form (Pusch et al., 1998; Tristani-Firouzi and Sanguinetti, 1998):

*model 1*:


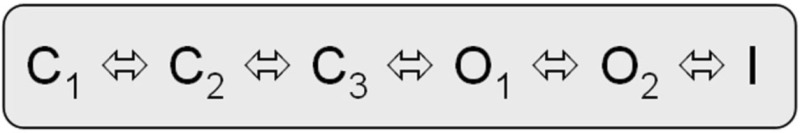


In this model C_1_ 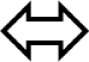 C_2_, C_2_ 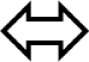 C_3_, and O_1_ 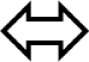 O_2_ are the voltage-dependent gating steps. Recently, an alternative circular gating scheme was proposed by Ma et al. (2011).

*model 2*:


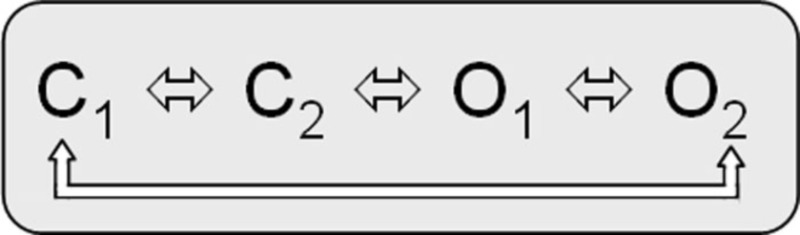


This novel gating scheme accounts for an open fraction that is determined by allosteric coupling of residues in the S4–S5 linker/pore domain (PD). C_1_ 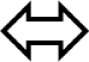 C_2_ and O_1_ 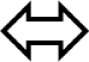 O_2_ are the voltage-dependent gating steps.

Kv7.1/KCNE1 heteromeric channels activate far more slowly and at more positive potentials than homomeric Kv7.1 channels, do not partially inactivate, and have an increased single-channel conductance compared to homomeric Kv7.1 recorded in standard extracellular solutions (Barhanin et al., 1996; Sanguinetti et al., 1996; Pusch, 1998; Pusch et al., 1998; Tristani-Firouzi and Sanguinetti, 1998). It is believed that Kv7.1/KCNE1 channel currents resemble the cardiac repolarizing, slowly activating potassium current *I*_Ks_ and that LQTS1/5 mutations might directly or allosterically modify interactions of Kv7.1 and the β-subunit KCNE1 (e.g., Schmitt et al., 2000; Seebohm et al., 2001c; Osteen et al., 2010; Wang et al., 2011b). Cui et al. (1994) proposed two linear-branched gating schemes for Kv7.1/KCNE1 heteromers, which depend on varying KCNE1 amounts coexpressed with Kv7.1 and imply interactions among individual channel proteins during activation. Yet Tzounopoulos et al. reported on a crossover gating phenomenon after prepulsing to different voltages that cannot be explained by a classical *Cole–Moore* shift in a linear gating model of Kv7.1/KCNE1 channels and presented results that argue for a circular gating model similar to model 2 (Cole and Moore, 1960; Tzounopoulos et al., 1998). However, a branched model can account for this crossover gating phenomenon after prepulsing to different voltages as well (Strutz-Seebohm et al., 2011):

*model 3*:


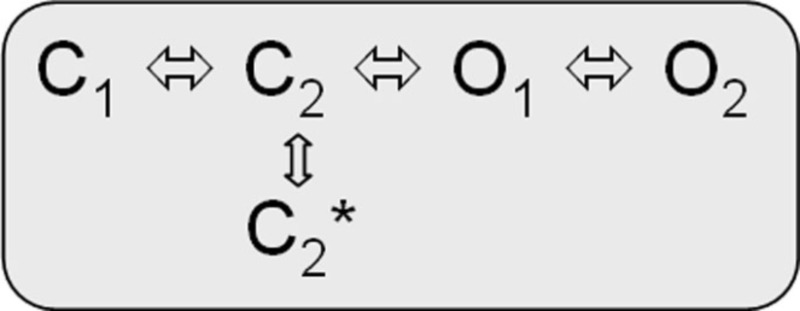


Further gating schemes have been proposed to describe Kv7.1/KCNE1 gating (Silva and Rudy, 2005, 2010; Osteen et al., 2010; Ghosh et al., 2011). In summary, the gating behaviors of Kv7.1 and Kv7.1/KCNE1 are relatively complex, and simple, linear gating models reach too short to approximate a realistic mathematical description.

## Stoichiometry of the Kv7.1/KCNE1 Complex

**Figure 3:**
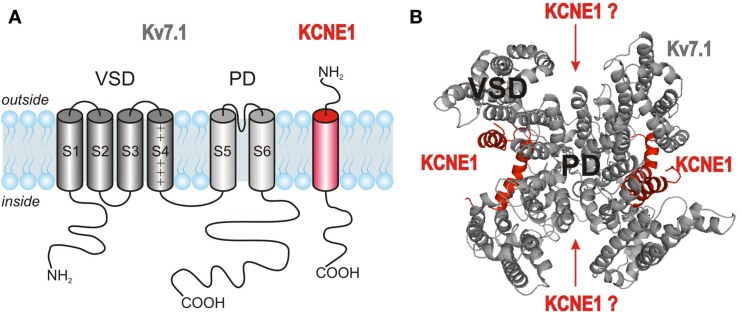
**Topology and stoichiometry of Kv7.1/KCNE1 channels**. **(A)** Kv7.1 α-subunits are made of six membrane-spanning segments S1–S6 and intracellular N- and C-terminal domains. The segments S1–S4 form the voltage-sensing domain (VSD), while the pore domain (PD) consists of segments S5 and S6. KCNE1 β-subunits contain a single transmembrane segment flanked by an extracellular N-terminus and a cytosolic C-terminus. **(B)** Four Kv7.1 α-subunits assemble to form a functional channel. The number of KCNE1 β-subunits associating with Kv7.1 tetramers is still a matter of extensive debate (Strutz-Seebohm et al., 2011).

After the discovery that the channel complex mediating the cardiac *I*_Ks_ is composed of Kv7.1 α-subunits and KCNE1 β-subunits (Barhanin et al., 1996; Sanguinetti et al., 1996), detailed investigations on the nature of this association started. Like all members of the Kv channel family, Kv7.1 α-subunits assemble into tetramers to form functional channels. Each α-subunit consists of six transmembrane segments, S1–S6, flanked by intracellular amino- and carboxy-terminal domains. The central pore domain (PD) of the channel, which contains the ion conduction pathway and the activation gate, is formed by segments S5 and S6 and the pore loop between them. The pore loop carries the K^+^ channel signature sequence TTIGYGD allowing rapid ion conduction and potassium selectivity. The PD is surrounded and controlled by four voltage-sensing domains (VSDs) formed by segments S1–S4 from each subunit (Figures 3A,B). S4 contains several positively charged amino acids and is a key element of voltage sensing. Further charged residues in S1–S3 may contribute additional charge to allow for voltage sensing. Tetrameric assembly of Kv7.1 α-subunits is mediated via their C-terminal domains (Schmitt et al., 2000; Schwake et al., 2003). Although Kv7.1 homotetramers are functional, the presence of the accessory β-subunit KCNE1 is needed to produce currents with characteristics of native *I*_Ks_ (Figure 2A). KCNE1 is a small, integral membrane protein with a single transmembrane-spanning segment flanked by an extracellular N-terminal and an intracellular C-terminal domain (Figure 3A). The number of KCNE1 β-subunits recruited by tetrameric Kv7.1 channels has been a matter of extensive debate (Figure 3B). Previous studies have either suggested a fixed stoichiometry of four Kv7.1 subunits and two KCNE1 subunits (Wang and Goldstein, 1995; Chen et al., 2003a; Kang et al., 2008; Morin and Kobertz, 2008) or a variable stoichiometry with up to four KCNE1 subunits assembling with a Kv7.1 tetramer (Cui et al., 1994; Wang et al., 1998, 2011a; Morokuma et al., 2008; Nakajo et al., 2010; Zheng et al., 2010; Strutz-Seebohm et al., 2011). A summary of the data obtained during the last two decades is provided in Table 1. The results reported leave the possibility open that KCNE1 subunits coassemble with Kv7.1 in different modes allowing up to four KCNE1 subunits to approach the Kv7.1 tetramer but permitting only formation of 4 Kv7.1 : 2 KCNE1 assemblies in specific regions of the heteromeric channels. Prerequisites for such an interaction would be variable and dynamic interactions. Indeed, several studies suggest interactions of Kv7.1 with KCNE1 at different protein regions in the various channel states (summarized in Table 2). However, further studies are required to support such a hypothesis.

**Table 1:** **Stoichiometry of Kv7.1/KCNE1 channels**.

Experimental evidence leading to conclusion of fixed stoichiometry (4:2)	Reference
Suppression of current induced by coexpression of wildtype and mutant KCNE1 indicates 4:2 stoichiometry	Wang and Goldstein (1995)
Kv7.1/Kv7.1/KCNE1 fusion proteins and naturally assembled Kv7.1/KCNE1 channels show similar characteristics of CTX inhibition; quantification of Kv7.1 and KCNE1 subunits using ^3^H-CTX and an antibody indicates a 4:2 stoichiometry	Chen et al. (2003a)
Chemical subunit counting experiments indicate that association of two KCNE1 subunits with the Kv7.1 tetramer is sufficient to induce KCNE1-typical modulation of channel properties	Morin and Kobertz (2008)
Computational model of Kv7.1/KCNE1 channels indicates that binding of more than two KCNE1 subunits to the Kv7.1 tetramer might be sterically hindered	Kang et al. (2008)

**Experimental evidence leading to conclusion of variable stoichiometry**	**Reference**

Current amplitude, activation kinetics, and voltage dependence of Kv7.1/KCNE1 channels vary with the amount of KCNE1	Cui et al. (1994)
Both Kv7.1/KCNE1 fusion proteins and Kv7.1–Kv7.1 with additional KCNE1 produce currents with activation kinetics and voltage dependence similar to naturally assembled Kv7.1/KCNE1 channels	Wang et al. (1998)
Voltage dependence of activation of Kv7.1/KCNE1 channels varies with the amount of KCNE1	Morokuma et al. (2008)
Single-molecule fluorescent bleaching studies indicate that up to four KCNE1 subunits associate with the Kv7.1 tetramer depending on the relative densities of the two subunits	Nakajo et al. (2010)
Effects of free KCNE1 C-terminals on voltage dependence of activation of Kv7.1 and Kv7.1/KCNE1 channels are complex, indicating multiple stoichiometries or saturation of possible binding sites	Zheng et al. (2010)
Overexpression of KCNE1 markedly changed activation kinetics and voltage dependence of native *I*_Ks_, indicating assembly of additional KCNE1 subunits with endogenous channels	Wang et al. (2011a)
Modeling of transmembrane domain suggests that variable stoichiometry seems possible in this region	Strutz-Seebohm et al. (2011)

**Table 2:** **Structural basis of Kv7.1/KCNE1 interaction**.

Approach	Conclusion	Reference
Deletion analysis, chimeric approach, and/or site-directed mutagenesis	TM segment and cytoplasmic portion immediately following TM segment of KCNE1 mediate KCNE1 function	Takumi et al. (1991)
	TM segment and C-terminal domain of KCNE1 mediate KCNE1 function	Tapper and George (2000)
	Residues 57–59 of KCNE1 are important for KCNE1 function (“activation triplet”)	Melman et al. (2001)
	Residue L273 of Kv7.1 is important for normal modulation by KCNE1	Seebohm et al. (2001c)
	Residue T58 is a key element of KCNE1 function	Melman et al. (2002)
	Requirements to interact with KCNE1 are located in regions C-terminal to S5; Residues S338, F339, and F340 in S6 are important for normal modulation by KCNE1	Melman et al. (2004)
	Residues S338, F339, and F340 in S6 are possible interaction sites of KCNE1	Panaghie et al. (2006)
	KCNE1 C-terminus is crucial for channel assembly, open state destabilization, kinetics of deactivation	Chen et al. (2009)
	Amino acids important for normal modulation by KCNE1 are located in S5 and S6 (G272, V324, V334) of Kv7.1	Nakajo et al. (2011)
Cysteine scanning mutagenesis combined with chemical modifications (Cd^2+^ coordination, MTS reagent binding, and spontaneous disulfide formation)	TM segment of KCNE1 lines the conduction pathway	Wang et al. (1996a)
	TM segment of KCNE1 lines the conduction pathway	Tai and Goldstein (1998)
	KCNE1 is located outside the conduction pathway	Kurokawa et al. (2001)
	KCNE1 is located outside the conduction pathway but in very close proximity to S6 of Kv7.1	Tapper and George (2001)
	E44 in KCNE1 is close to A226 in S4 of Kv7.1 in the open state; KCNE1 is close to or possibly interacts with the VSD	Nakajo and Kubo (2007)
	KCNE1 makes state-dependent contact with S1 of Kv7.1; KCNE1 is in close proximity to the VSD	Xu et al. (2008)
	KCNE1 is located close to S1 and S4 of two adjacent VSDs	Shamgar et al. (2008)
	C-terminal region directly following the KCNE1 TM segment interacts with activation gate of Kv7.1	Lvov et al. (2010)
Proof of direct physical interaction or close proximity (e.g., co-immunoprecipitation, FRET)	C-terminus of KCNE1 directly interacts with the pore region of Kv7.1	Romey et al. (1997)
	C-termini of Kv7.1 and KCNE1 move close to each other during channel activation; distal C-terminus of KCNE1 interacts with dimeric coiled coil helix C of Kv7.1	Haitin et al. (2009)
	Physical interaction between C-termini of Kv7.1 and KCNE1; portion of A-helix and its linker to S6 bind KCNE1	Zheng et al. (2010)
Analysis of disease-causing mutations	Functional interaction of KCNE1 with S4/S4–S5 linker of Kv7.1	Franqueza et al. (1999)
	Functional interaction of KCNE1 with S4/S4–S5 linker of Kv7.1	Chouabe et al. (2000)
	Functional interaction of KCNE1 with S1 of Kv7.1	Chan et al. (2012)
Computational model	KCNE1 is located in a cleft between the pore domain and the VSD of Kv7.1	Kang et al. (2008)
	KCNE1 binds to the outer face of the Kv7.1 channel pore, KCNE1 is located in a cleft between pore domain and VSD of Kv7.1	Strutz-Seebohm et al. (2011)

## Structural Basis of Kv7.1/KCNE1 Interaction

The structural basis of the Kv7.1/KCNE1 interaction has been studied extensively over the last 20 years. Experimental data obtained from deletion analysis, chimeric approaches, and site-directed mutagenesis identified regions in both Kv7.1 and KCNE1 that might be crucial for association and modulation (summarized in Table 2). Based on these data it has been suggested that the transmembrane segment and the C-terminal domain of KCNE1 mediate its ability to modulate Kv7.1 (Takumi et al., 1991; Tapper and George, 2000). Molecular key elements of KCNE1 function seem to be three glycines, several bulky, aromatic phenylalanines, and the threonine 58 located at the center of the KCNE1 transmembrane segment (Melman et al., 2001, 2002; Strutz-Seebohm et al., 2011). A comparison with other KCNE subunits underscores the importance of the transmembrane segment for KCNE function: interactions of Kv7.1 with KCNE1 and Kv7.1 with KCNE3 show similar chemical modification rates within the transmembrane region (Rocheleau and Kobertz, 2008), and the KCNE4 transmembrane segment modulates voltage dependence of Kv7.1 activation (Ciampa et al., 2011). A recent study indicates that different KCNE proteins contact different regions in Kv7.1 all located within the transmembrane segments (Nakajo et al., 2011). In segment S6 of Kv7.1, a three amino acid motif (S338, F339, F340) has been identified that might constitute a site of specific interaction with KCNE1, especially with its residue T58 (Melman et al., 2004; Panaghie et al., 2006; Strutz-Seebohm et al., 2011). Further residues in the pore region of Kv7.1 (F270, G272, and L273 in S5, V307, V310, and T311 in the lower pore helix, and V324 and V334 in the upper S6) are crucial for an accurate modulation by KCNE1 (Seebohm et al., 2001c, 2003c; Nakajo et al., 2011).

Based on experimental evidence, localizations of KCNE1 in the channel complex have been suggested. A series of observations obtained from cysteine scanning mutagenesis combined with chemical modifications (Cd^2+^ coordination, MTS reagent binding, and spontaneous disulfide formation) indicate that KCNE1 lies in close proximity to both the PD and the VSD of the Kv7.1 tetramer (summarized in Table 2). First interpretations suggested that KCNE1 lines the conduction pathway (Wang et al., 1996a; Tai and Goldstein, 1998). However, subsequent studies indicate that the KCNE1 transmembrane segment is located outside the PD and interacts with the outer S5 and S6 segments of Kv7.1 (Lerche et al., 2000; Kurokawa et al., 2001; Tapper and George, 2001; Chung et al., 2009; Strutz-Seebohm et al., 2011). Its extracellular flank seems to face the extracellular ends of segments S1 and S6 of Kv7.1, with the residues interacting with segment S1 varying dependent on the specific channel state (Xu et al., 2008; Chung et al., 2009). In addition to segment S1 of the VSD and S5/S6 of the PD, the transmembrane domain of KCNE1 gets in close contact with the primary cationic voltage sensor S4 (Nakajo and Kubo, 2007; Shamgar et al., 2008; Silva et al., 2009; Strutz-Seebohm et al., 2011). A possible interaction of KCNE1 with the VSD of Kv7.1 is further underscored by the fact that the abnormal phenotypes of several disease-causing mutations in S1, S4, or the S4–S5 linker of Kv7.1 *in vitro* only manifest in the presence of KCNE1 (summarized in Table 2; Franqueza et al., 1999; Chouabe et al., 2000; Chen et al., 2003c; Chan et al., 2012). The cytoplasmic portion directly following the transmembrane segment of KCNE1 seems to interact with the gating machinery of Kv7.1 (Lvov et al., 2010). A hallmark of all Kv7 channels is their sensitivity to muscarinic signaling, which gave them the name M-channels. The Kv7.1 membrane-proximal region may link membrane lipid metabolism of phosphoinositide PI(4,5)P_2_ to the gating machinery to allow for integration of muscarinic signaling into gating alterations in Kv7.1 (Ikeda and Kammermeier, 2002; Loussouarn et al., 2003). The lower S6 is expected to be in close proximity to allow for electrostatic interaction with the PI(4,5)P_2_ head groups, and PI(4,5)P_2_ binding sites have been reported to be positioned close to the lower S6-helices bundle in the PD of Kv7.1 (Loussouarn et al., 2003; Thomas et al., 2011). However, it may be mentioned that further PI(4,5)P_2_ binding sites may exist in the TM domain. KCNE1 residues R^67^SKKLEH^73^ in the vicinity of the inner membrane leaflet increase the PI(4,5)P_2_ sensitivity of Kv7.1 by a factor of 100, possibly by electrostatic interaction with PI(4,5)P_2_ head groups and/or Kv7.1 residues interacting with PI(4,5)P_2_ (Li et al., 2011). Thus, PI(4,5)P_2_ may function as a molecular coupler of Kv7.1 and KCNE1 to modulate gating in complex ways.

Recent structural modeling approaches generated 3D models of the transmembrane region of Kv7.1/KCNE1 that are in large agreement with the various experimental data. Specifically, homology modeling of the Kv7.1 S1–S6 region and *Rosetta*-based docking of the KCNE1 transmembrane domain produced an open and a closed state model of the heteromeric channel complex (Smith et al., 2007; Kang et al., 2008). Very recently, homology modeling of Kv7.1 with manual docking of the KCNE1 transmembrane domain was used to generate a pre-open closed state model of the Kv7.1/KCNE1 channel (Strutz-Seebohm et al., 2011). All three models are relatively stable in molecular dynamics simulations, which suggests that they are close to native states.

The C-termini of Kv7.1 and KCNE1 are in close proximity and physically interact, as indicated by FRET measurements and co-immunoprecipitation, yet the structural basis of this interaction is unknown (Haitin et al., 2009; Zheng et al., 2010). The cytosolic domain of Kv7.1/KCNE1 channels represents a multi-modular structure serving multiple functions. It contains several amphipathic α-helices. Structural prediction algorithms place two α-helices, A and B, to the proximal C-terminus of Kv7.1 (Haitin and Attali, 2008). These α-helices may contain functionally relevant calmodulin interaction motifs. The residues Kv7.1^586–618^ form α-helix D that contain a leucine-zipper and a coiled coil fold (Kanki et al., 2004). This parallel, four-stranded coiled coil allows for specific tetrameric assembly of Kv7.1 channel subunits (Schmitt et al., 2000; Schwake et al., 2003; Nakajo and Kubo, 2008). Recently, Xu and Minor (2009) presented a high-resolution X-ray crystal structure (1.7 Å resolution) of Kv7.1^583–611^ confirming the predictions of the coiled coil assembly specificity domain. Interestingly, helix D is supposedly two helical turns longer in this crystal structure than helices D of the closely related Kv7.2–5 channels, which allows for specific homomeric assembly with other Kv7.1 subunits but not for heteromeric assemblies with other family members (Haitin and Attali, 2008; Xu and Minor, 2009). The S6 transmembrane segment and the A-domain are linked by the region Kv7.1^350–582^, which according to *in silico* prediction assumes an apo-chloroperoxidase fold and can thus be modeled using the structural coordinates of apo-chloroperoxidase (pdb: 1VNS; Macedo-Ribeiro et al., 1999). However, these predictions appear somewhat preliminary, and several assumptions have to be made to incorporate the modeled region into the rest of the Kv7.1–3D model. Further, how exactly the proposed helices A/B and linker region containing the calmodulin binding site can be integrated into this fold is speculative. The structural folds of the N-terminal region Kv7.1^1–115^ and the most distal residues Kv7.1^612–676^ remain completely unknown.

PI(4,5)P_2_ modulates function of Kv7.1/KCNE1 channels, and functional PI(4,5)P_2_ interaction sites were reported not only for the lower S6 segment but also for the C-terminus, where a cluster of basic amino acids may form a PI(4,5)P_2_ interaction site allowing for electrostatic interactions with the PI(4,5)P_2_ head groups (Loussouarn et al., 2003; Zhang et al., 2003; Delmas and Brown, 2005). This PI(4,5)P_2_ interaction site may functionally and physically overlap with the calmodulin binding sites in the inter helix A/B linker region (Hernandez et al., 2008). The Kv7.1–calmodulin interaction can be modulated by coassembly with KCNE4. This modulation is dependent on a juxtamembrane tetra-leucine motif (L69–L72) in KCNE4. However, deletion of this motif leaves the kinetic effects on Kv7.1 mediated by the KCNE4 transmembrane domain intact (Ciampa et al., 2011). Possibly, PI(4,5)P_2_ and calmodulin “glue” these C-terminal regions to the transmembrane proximal regions mentioned above and allow for modulation of gating at the activation gate and the VSD. Clearly, further evidence is needed to prove this hypothesis (Li et al., 2011).

**Figure 4:**
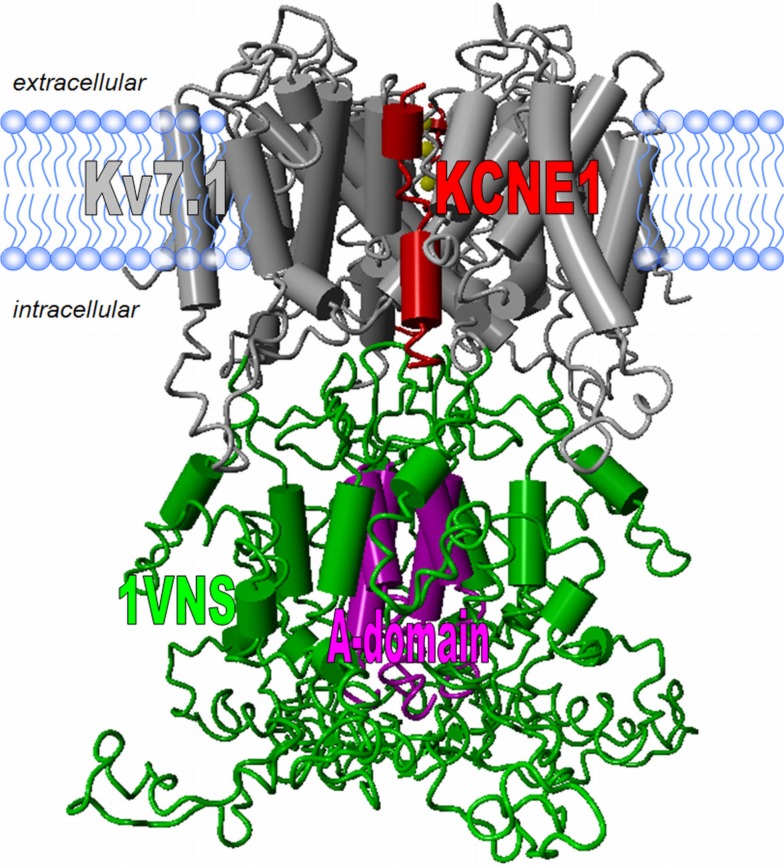
**Structural model of the Kv7.1 channel**. The transmembrane domain of the Kv7.1 channel bears the common Kv channel structure. Homology models using the solved crystal structural constraints allow for good model predictions (gray). For docking experiments the solved NMR-structural data of the full-length KCNE1 in lipid environments can be used (here only the transmembrane segment is shown in red; Tian et al., 2007; Kang et al., 2008). The structure of the Kv7.1 tetrameric assembly domain (A-domain) was solved and can be incorporated into a model (Xu and Minor, 2009). The region linking the S6 and the A-domain shows amino acid similarity to the structure 1VNS.pdb. No structures or even template coordinates for the Kv7.1 N-terminus and the distal C-terminus are available and thus homology modeling is currently problematic.

The C-termini of Kv7.1/KCNE1 channels physically interact with several proteins like the AKAP yotiao, epidermal growth factor receptor kinase, and NEDD4.2 (Marx et al., 2002; Kanki et al., 2004; Kurokawa et al., 2004; Jespersen et al., 2007; Dong et al., 2010). The protein kinase A phosphorylates Ser27 in the N-terminus of Kv7.1 and modulates Kv7.1/KCNE1 function (Marx et al., 2002). This modulation critically depends on the presence of KCNE1 (Marx et al., 2002; Kurokawa et al., 2004). This observation suggests that the Kv7.1 N-terminus contributes to Kv7.1/KCNE1 subunit interaction. The binding sites for these proteins have to be located at the surface of the C-terminal structure to be accessible for interaction. This information will be of help for further structural modeling. A 3D model of the Kv7.1/KCNE1 channel satisfying most of the aforementioned hypotheses is shown in Figure 4.

## Toward a Dynamic View on Structural Rearrangements during Kv7.1/KCNE1 Gating

In the last 14 years a wealth of potassium channel crystal structures have been reported. Although central structural elements in Kv7.1 clearly differ from classical Shaker-type Kv channels, the available crystal structures are well-suited templates to model the Kv7.1 S1–S6 region in different states (Seebohm et al., 2006; Silva et al., 2009). Direct interactions of the transmembrane segments of Kv7.1 with KCNE1 have been proposed, but the specific amino acid interactions remain controversial (summarized in Table 2). CD spectra in a membrane-like environment and the NMR solution structure of KCNE1 transmembrane peptides show that this peptide adopts an α-helical structure (Aggeli et al., 1998; Strutz-Seebohm et al., 2011). Recently, two NMR structures of full-length KCNE1 confirmed that the transmembrane segment of KCNE1 folds an α-helix (Tian et al., 2007; Kang et al., 2008). An open and a closed state model of Kv7.1/KCNE1 channels were proposed based on the NMR-structural coordinates and *Rosetta*-dockings of KCNE1 to Kv7.1 that suggest binding of KCNE1 to the so-called “gain-of-function cleft”, a space formed between two adjacent voltage sensor domains and the outer PD (Smith et al., 2007; Kang et al., 2008).

**Figure 5:**
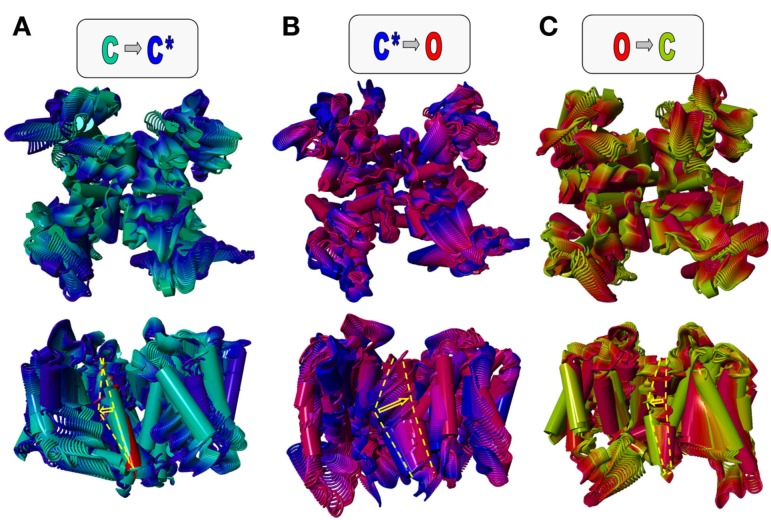
**Dynamic model of gating transitions in Kv7.1/KCNE1 channels**. The closed (C), pre-open (C*), and open (O) state models described by Smith et al. (2007) and Strutz-Seebohm et al. (2011) were used to generate dynamic models using simple morphing approaches. The gating of Kv7.1/KCNE1 channels can be approximated by a simplified circular gating model. Major gating steps are C ⇒ C* **(A)**, C* ⇒ O **(B)**, and O ⇒ C **(C)** (colors as indicated in the letters above the models). For clarity the central axes of the KCNE1 start and end models are marked by the yellow dashed lines, and the direction and extent of the proposed motion is indicated by the yellow arrow. In **(A)** the KCNE1 start model is shown in red and the end model in magenta. The morphs indicate larger motions of KCNE1 during channel gating in the gating cleft of Kv7.1 channels. A particularly large gating motion around Thr58 is seen from C* ⇒ O **(B)**, suggesting a major energetic barrier. This may be the molecular basis of the dramatically slowed activation of Kv7.1 by association with KCNE1. However, calculation of energies on the models is highly speculative because specific interactions with surrounding membrane molecules [e.g., PI(4,5)P_2_] would be highly influential, and the current knowledge on lipid-channel interactions is not sufficient to allow for precise calculations.

Localized to the center of the α-helical KCNE1 transmembrane segment are the residues G^52^FFGFFTLG^60^. As predicted, this α-helical transmembrane domain allows for flexibility at glycine residues G52, G55, and G60 (Tian et al., 2007; Kang et al., 2008; Strutz-Seebohm et al., 2011). Key to functional modulation of Kv7.1 by KCNE1 is the residue T58 (Melman et al., 2002). The surrounding phenylalanine residues generate a bulky aromatic cuff. The combination of these structural elements may enable formation of a stable hydrogen bond with residue S338 in Kv7.1 and diverse van der Waals interactions with residues in S4 and the S4–S5 linker region as well as S5/S6 amino acids F270, F339, and F340 to stabilize a pre-open closed state. The combination of the Kv7.1/KCNE1 models, the closed state, the pre-open closed state and the open state model are in good agreement with published results of several experimental studies (Smith et al., 2007; Kang et al., 2008; Strutz-Seebohm et al., 2011). The modeled transmembrane domain structures can be used to assess a molecular view on the slow activation of *I*_Ks_ (Figure 5). The three states closed, pre-open, and open can be used for morphing calculations (Echols et al., 2003). The basic idea is that the channel undergoes conformational transitions from closed (C) to pre-open (C^*^), from pre-open (C^*^) to open (O), and from open (O) to closed (C) states. These seem to represent the major steps in the Kv7.1 and Kv7.1/KCNE1 gating (Silva et al., 2009). The position of the voltage sensor is “down” for the closed state and “up” for the pre-open and open states (Tian et al., 2007; Kang et al., 2008; Silva et al., 2009; Strutz-Seebohm et al., 2011). The interaction of KCNE1^T58^ with Kv7.1^S338^ and Kv7.1^F339^ may uncouple the gate at the lower S6 from the S4–S5 linker, which leads to S6 bundle crossing and closure of the gate (Strutz-Seebohm et al., 2011). The formation of the S6 bundle upon S4–S5 linker uncoupling may be supported by the lack of a central S6 gating hinge (Seebohm et al., 2006). The uncoupling of S4–S5 by KCNE1 during closed state inactivation shows similarities to an uncoupling event of the PD from the voltage sensor domain during closed state inactivation of some Kv channels as described by the group of Bähring et al. (2012). In the light of the ongoing discussion about fixed stoichiometry of two KCNE1 subunits per Kv7.1 tetramer vs. variable stoichiometry it is fair to study a channel complex with two KCNE1 subunits docked to four Kv7.1 subunits (Strutz-Seebohm et al., 2011). This stoichiometry is consistent with both hypotheses. Morphing experiments have been performed (Figure 5). As expected the gating is associated with large structural rearrangements in the voltage sensor domain. On the other hand the PD is rather rigid in the region around the selectivity filter. The KCNE1 transmembrane segment undergoes relatively small rearrangements during the gating steps C 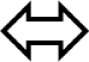 C^*^ and O 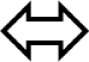 C but dramatic structural alterations during the C^*^ 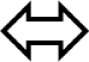 O transition (Figure 5; the extent of structural rearrangement around key residue KCNE1 T^58^ is indicated by a yellow arrow). This dramatic rearrangement of KCNE1 associated with the C^*^ 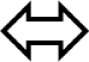 O transition may generate an energetic barrier that can only slowly be overcome, resulting in dramatically slowed activation, the pivotal characteristic of Kv7.1/KCNE1 gating. Clearly, these models present a preliminary view on the dynamics of Kv7.1/KCNE1 channels. Silva et al. (2009) modeled further structural closed states of the Kv7.1 voltage sensor module. Incorporation of these data in future Kv7.1/KCNE1 morphing experiments may prove valuable. Increase in knowledge combined with the already available data, upcoming improvements in computational methodology, and decreasing computational costs will allow for much more facilitated dynamic modeling of Kv7.1/KCNE1 channel gating in the future.

The novel structural “snapshots” allow for a better understanding of Kv7.1/KCNE1 molecular pharmacology. Several Kv7.1/KCNE1 inhibitors and activators bind to distinct sites in state-dependent manner (Lerche et al., 2000, 2007; Seebohm et al., 2001b, 2003a; Chen et al., 2003b). Docking of various compounds to the different models and experimental verifications are now possible. An interesting observation is that the KCNE1 transmembrane segment induces large fenestrations in the Kv7.1 channel pre-open state that may allow for a novel access pathway not present in Kv7.1 homomeric channels (Strutz-Seebohm et al., 2011). Affected by these structural effects is the central cavity, the binding and blocking site of several Kv7.1/KCNE1 inhibitors (Lerche et al., 2000, 2007; Seebohm et al., 2003a). This altered structure could be used as a scaffold for the computer-aided design of specific Kv7.1/KCNE1 modulators not targeting homomeric Kv7.1 channels, which would be a prerequisite for tissue specific pharmacology (Seebohm et al., 2003b; Strutz-Seebohm et al., 2011). Insights into KCNE1-specific molecular pharmacology will be highly valuable for both drug development and safety pharmacology alike (Towart et al., 2009; Pugsley et al., 2011).

Upcoming physiological, pathophysiological, pharmacological, and structural analyses will fill the gaps in our current knowledge, and therefore the old Kv7.1/KCNE1 channel faces an exciting future. Let’s dance the KCNQ1 tango!

## Conflict of Interest Statement

The authors declare that the research was conducted in the absence of any commercial or financial relationships that could be construed as a potential conflict of interest.

## Appendix

**Table A1:** **Kv7.1 expression in mammalian tissues**.

Specified tissue	Species	mRNA/protein	Reference
Brain	Mouse	mRNA	Lee et al. (2000)
	Mouse	mRNA	Ohya et al. (2003)
	Mouse	mRNA	Yeung et al. (2007)
	Mouse	mRNA	Iannotti et al. (2010)
Inner ear	Mouse	mRNA	Neyroud et al. (1997)
	Mouse	mRNA/protein	Nicolas et al. (2001)
	Mouse	Protein	Knipper et al. (2006)
	Rat	mRNA	Liang et al. (2006)
	Rat	Protein	Hur et al. (2007)
	Guinea pig	mRNA	Liang et al. (2006)
Trachea	Mouse	mRNA/protein	Grahammer et al. (2001b)
Thyroid gland	Mouse	mRNA/protein	Frohlich et al. (2011)
	Human	mRNA	Yang et al. (1997)
	Human	mRNA	Frohlich et al. (2011)
Thymus	Mouse	mRNA	Demolombe et al. (2001)
	Human	mRNA	Chouabe et al. (1997)
Lung	Mouse	mRNA	Demolombe et al. (2001)
	Human	mRNA	Wang et al. (1996b)
	Human	mRNA	Sanguinetti et al. (1996)
	Human	mRNA	Chouabe et al. (1997)
	Human	mRNA	Yang et al. (1997)
Heart	Mouse	mRNA	Barhanin et al. (1996)
	Mouse	mRNA	Lee et al. (2000)
	Mouse	mRNA	Casimiro et al. (2001)
	Mouse	mRNA	Demolombe et al. (2001)
	Mouse	mRNA	Ohya et al. (2003)
	Mouse	mRNA	Yeung et al. (2007)
	Mouse	mRNA	Iannotti et al. (2010)
	Mouse	mRNA	Strutz-Seebohm et al. (2006)
	Mouse	Protein	Knollmann et al. (2007)
	Rat	Protein	Rasmussen et al. (2004)
	Guinea pig	mRNA/protein	Zicha et al. (2003)
	Guinea pig	Protein	Nicolas et al. (2008)
	Ferret	mRNA	Brahmajothi et al. (1997)
	Rabbit	mRNA/protein	Zicha et al. (2003)
	Horse	Protein	Finley et al. (2002)
	Canine	mRNA/protein	Han et al. (2002)
	Human	mRNA	Wang et al. (1996b)
	Human	mRNA	Sanguinetti et al. (1996)
	Human	mRNA	Chouabe et al. (1997)
	Human	mRNA	Yang et al. (1997)
	Human	mRNA	Bendahhou et al. (2005)
	Human	mRNA	Gaborit et al. (2007)
	Human	mRNA	Frohlich et al. (2011)
	Human	mRNA/protein	Zicha et al. (2003)
Liver	Mouse	mRNA	Lee et al. (2000)
	Mouse	mRNA	Demolombe et al. (2001)
Spleen	Mouse	mRNA	Demolombe et al. (2001)
	Human	mRNA	Chouabe et al. (1997)
Pancreas	Mouse	mRNA	Demolombe et al. (2001)
	Rat	Protein	Lee et al. (2008)
	Human	mRNA	Sanguinetti et al. (1996)
	Human	mRNA	Chouabe et al. (1997)
	Human	mRNA	Yang et al. (1997)
Stomach	Mouse	mRNA	Lee et al. (2000)
	Mouse	mRNA	Demolombe et al. (2001)
	Mouse	mRNA/protein	Dedek and Waldegger (2001)
	Mouse	Protein	Grahammer et al. (2001a)
	Mouse	Protein	Heitzmann et al. (2004)
	Mouse	Protein	Heitzmann and Warth (2007)
	Rat	mRNA/protein	Lambrecht et al. (2005)
	Human	mRNA	Yang et al. (1997)
	Human	mRNA	Frohlich et al. (2011)
	Human	Protein	Grahammer et al. (2001a)
Colon	Mouse	mRNA/protein	Dedek and Waldegger (2001)
	Rat	mRNA	Kunzelmann et al. (2001)
	Human	mRNA	Chouabe et al. (1997)
	Human	mRNA	Yang et al. (1997)
	Human	mRNA	Frohlich et al. (2011)
	Human	mRNA/protein	Horikawa et al. (2005)
Small intestine	Mouse	mRNA	Demolombe et al. (2001)
	Mouse	mRNA/protein	Dedek and Waldegger (2001)
	Human	mRNA	Chouabe et al. (1997)
	Human	mRNA	Yang et al. (1997)
Kidney	Mouse	mRNA	Barhanin et al. (1996)
	Mouse	mRNA	Lee et al. (2000)
	Mouse	mRNA	Demolombe et al. (2001)
	Mouse	Protein	Vallon et al. (2001)
	Human	mRNA	Wang et al. (1996b)
	Human	mRNA	Sanguinetti et al. (1996)
	Human	mRNA	Chouabe et al. (1997)
	Human	mRNA	Yang et al. (1997)
Uterus	Mouse	mRNA/protein	McCallum et al. (2009)
Placenta	Human	mRNA	Wang et al. (1996b)
	Human	mRNA	Sanguinetti et al. (1996)
	Human	mRNA	Chouabe et al. (1997)
	Human	mRNA	Yang et al. (1997)
Ovary	Human	mRNA	Chouabe et al. (1997)
Prostate	Human	mRNA	Chouabe et al. (1997)
	Human	mRNA	Yang et al. (1997)
Testis	Human	mRNA	Chouabe et al. (1997)
Blood vessels	Mouse	mRNA/protein	Ohya et al. (2003)
	Mouse	mRNA/protein	Yeung et al. (2007)
	Rat	mRNA	Brueggemann et al. (2007)
	Rat	mRNA	Mackie et al. (2008)
	Rat	mRNA	Joshi et al. (2009)
	Rat	mRNA/protein	Zhong et al. (2010)
	Human	mRNA/protein	Ng et al. (2011)
Skeletal muscle	Mouse	mRNA	Demolombe et al. (2001)
	Mouse	mRNA	Iannotti et al. (2010)
	Rat	mRNA	Roura-Ferrer et al. (2008)
Peripheral blood leukocytes	Human	mRNA	Chouabe et al. (1997)
Hematopoietic stem cells	Human	mRNA	Park et al. (2011)

## References

[B1] Abbott G. W. Sesti F. Splawski I. Buck M. E. Lehmann M. H. Timothy K. W. Keating M. T. Goldstein S. A. (1999). MiRP1 forms IKr potassium channels with HERG and is associated with cardiac arrhythmia. Cell 97, 175–187 10.1016/S0092-8674(00)80728-X 10219239

[B2] Aggeli A. Bannister M. L. Bell M. Boden N. Findlay J. B. Hunter M. Knowles P. F. Yang J. C. (1998). Conformation and ion-channeling activity of a 27-residue peptide modeled on the single-transmembrane segment of the IsK (minK) protein. Biochemistry 37, 8121–8131 10.1021/bi972112h 9609707

[B3] Angelo K. Jespersen T. Grunnet M. Nielsen M. S. Klaerke D. A. Olesen S. P. (2002). KCNE5 induces time- and voltage-dependent modulation of the KCNQ1 current. Biophys. J. 83, 1997–2006 10.1016/S0006-3495(02)73961-1 12324418 PMC1302289

[B4] Arrighi I. Bloch-Faure M. Grahammer F. Bleich M. Warth R. Mengual R. Drici M. D. Barhanin J. Meneton P. (2001). Altered potassium balance and aldosterone secretion in a mouse model of human congenital long QT syndrome. Proc. Natl. Acad. Sci. U.S.A. 98, 8792–8797 10.1073/pnas.141233398 11438691 PMC37514

[B5] Bähring R. Barghaan J. Westermeier R. Wollberg J. (2012). Voltage sensor inactivation in potassium channels. Front. Pharmacol. 3:100 10.3389/fphar.2012.00100 22654758 PMC3358694

[B6] Barhanin J. Lesage F. Guillemare E. Fink M. Lazdunski M. Romey G. (1996). K(V)LQT1 and lsK (minK) proteins associate to form the I(Ks) cardiac potassium current. Nature 384, 78–80 10.1038/384078a0 8900282

[B7] Bendahhou S. Marionneau C. Haurogne K. Larroque M. M. Derand R. Szuts V. Escande D. Demolombe S. Barhanin J. (2005). In vitro molecular interactions and distribution of KCNE family with KCNQ1 in the human heart. Cardiovasc. Res. 67, 529–538 10.1016/j.cardiores.2005.02.014 16039274

[B8] Brahmajothi M. V. Morales M. J. Rasmusson R. L. Campbell D. L. Strauss H. C. (1997). Heterogeneity in K+ channel transcript expression detected in isolated ferret cardiac myocytes. Pacing Clin. Electrophysiol. 20, 388–396 10.1111/j.1540-8159.1997.tb06198.x 9058843

[B9] Brueggemann L. I. Moran C. J. Barakat J. A. Yeh J. Z. Cribbs L. L. Byron K. L. (2007). Vasopressin stimulates action potential firing by protein kinase C-dependent inhibition of KCNQ5 in A7r5 rat aortic smooth muscle cells. Am. J. Physiol. Heart Circ. Physiol. 292, H1352–H1363 10.1152/ajpheart.00065.2006 17071736 PMC2577603

[B10] Casimiro M. C. Knollmann B. C. Ebert S. N. Vary J. C. Jr. Greene A. E. Franz M. R. Grinberg A. Huang S. P. Pfeifer K. (2001). Targeted disruption of the Kcnq1 gene produces a mouse model of Jervell and Lange-Nielsen Syndrome. Proc. Natl. Acad. Sci. U.S.A. 98, 2526–2531 10.1073/pnas.041398998 11226272 PMC30171

[B11] Chadha P. S. Zunke F. Davis A. J. Jepps T. A. Linders J. T. Schwake M. Towart R. Greenwood I. A. (2012). Pharmacological dissection of K(v) 7.1 channels in systemic and pulmonary arteries. Br. J. Pharmacol. 166, 1377–1387 10.1111/j.1476-5381.2012.01863.x 22251082 PMC3417453

[B12] Chan P. J. Osteen J. D. Xiong D. Bohnen M. S. Doshi D. Sampson K. J. Marx S. O. Karlin A. Kass R. S. (2012). Characterization of KCNQ1 atrial fibrillation mutations reveals distinct dependence on KCNE1. J. Gen. Physiol. 139, 135–144 10.1085/jgp.201110672 22250012 PMC3269792

[B13] Chen H. Kim L. A. Rajan S. Xu S. Goldstein S. A. (2003a). Charybdotoxin binding in the I(Ks) pore demonstrates two MinK subunits in each channel complex. Neuron 40, 15–23 10.1016/S0896-6273(03)00570-1 14527430

[B14] Chen H. Sesti F. Goldstein S. A. (2003b). Pore- and state-dependent cadmium block of I(Ks) channels formed with MinK-55C and wild-type KCNQ1 subunits. Biophys. J. 84, 3679–3689 10.1016/S0006-3495(03)74998-4 12770875 PMC1302951

[B15] Chen Y. H. Xu S. J. Bendahhou S. Wang X. L. Wang Y. Xu W. Y. Jin H. W. Sun H. Su X. Y. Zhuang Q. N. Yang Y. Q. Li Y. B. Liu Y. Xu H. J. Li X. F. Ma N. Mou C. P. Chen Z. Barhanin J. Huang W. (2003c). KCNQ1 gain-of-function mutation in familial atrial fibrillation. Science 299, 251–254 10.1126/science.1080819 12522251

[B16] Chen J. Zheng R. Melman Y. F. McDonald T. V. (2009). Functional interactions between KCNE1 C-terminus and the KCNQ1 channel. PLoS ONE 4, e5143 10.1371/journal.pone.0005143 19340287 PMC2659744

[B17] Chouabe C. Neyroud N. Guicheney P. Lazdunski M. Romey G. Barhanin J. (1997). Properties of KvLQT1 K+ channel mutations in Romano-Ward and Jervell and Lange-Nielsen inherited cardiac arrhythmias. EMBO J. 16, 5472–5479 10.1093/emboj/16.17.5472 9312006 PMC1170178

[B18] Chouabe C. Neyroud N. Richard P. Denjoy I. Hainque B. Romey G. Drici M. D. Guicheney P. Barhanin J. (2000). Novel mutations in KvLQT1 that affect Iks activation through interactions with Isk. Cardiovasc. Res. 45, 971–980 10.1016/S0008-6363(99)00411-3 10728423

[B19] Chung D. Y. Chan P. J. Bankston J. R. Yang L. Liu G. Marx S. O. Karlin A. Kass R. S. (2009). Location of KCNE1 relative to KCNQ1 in the I(KS) potassium channel by disulfide cross-linking of substituted cysteines. Proc. Natl. Acad. Sci. U.S.A. 106, 743–748 10.1073/pnas.0911436106 19131515 PMC2630058

[B20] Ciampa E. J. Welch R. C. Vanoye C. G. George A. L. Jr. (2011). KCNE4 juxtamembrane region is required for interaction with calmodulin and for functional suppression of KCNQ1. J. Biol. Chem. 286, 4141–4149 10.1074/jbc.M110.158865 21118809 PMC3039368

[B21] Cole K. S. Moore J. W. (1960). Potassium ion current in the squid giant axon: dynamic characteristic. Biophys. J. 1, 1–14 10.1016/S0006-3495(60)86871-3 13694549 PMC1366308

[B22] Cui J. Kline R. P. Pennefather P. Cohen I. S. (1994). Gating of IsK expressed in *Xenopus oocytes* depends on the amount of mRNA injected. J. Gen. Physiol. 104, 87–105 10.1085/jgp.104.1.87 7964597 PMC2229196

[B23] Dedek K. Waldegger S. (2001). Colocalization of KCNQ1/KCNE channel subunits in the mouse gastrointestinal tract. Pflugers Arch. 442, 896–902 10.1007/s004240100609 11680623

[B24] Delmas P. Brown D. A. (2005). Pathways modulating neural KCNQ/M (Kv7) potassium channels. Nat. Rev. Neurosci. 6, 850–862 10.1038/nrm1746 16261179

[B25] Demolombe S. Franco D. de Boer P. Kuperschmidt S. Roden D. Pereon Y. Jarry A. Moorman A. F. Escande D. (2001). Differential expression of KvLQT1 and its regulator IsK in mouse epithelia. Am. J. Physiol., Cell Physiol. 280, C359–C372 11208532 10.1152/ajpcell.2001.280.2.C359

[B26] Dong M. Q. Sun H. Y. Tang Q. Tse H. F. Lau C. P. Li G. R. (2010). Regulation of human cardiac KCNQ1/KCNE1 channel by epidermal growth factor receptor kinase. Biochim. Biophys. Acta 1798, 995–1001 10.1016/j.bbamem.2010.01.010 20085748

[B27] Echols N. Milburn D. Gerstein M. (2003). MolMovDB: analysis and visualization of conformational change and structural flexibility. Nucleic Acids Res. 31, 478–482 10.1093/nar/gkg104 12520056 PMC165551

[B28] Finley M. R. Li Y. Hua F. Lillich J. Mitchell K. E. Ganta S. Gilmour R. F. Jr. Freeman L. C. (2002). Expression and coassociation of ERG1, KCNQ1, and KCNE1 potassium channel proteins in horse heart. Am. J. Physiol. Heart Circ. Physiol. 283, H126–H138 12063283 10.1152/ajpheart.00622.2001

[B29] Franqueza L. Lin M. Shen J. Splawski I. Keating M. T. Sanguinetti M. C. (1999). Long QT syndrome-associated mutations in the S4–S5 linker of KvLQT1 potassium channels modify gating and interaction with minK subunits. J. Biol. Chem. 274, 21063–21070 10.1074/jbc.274.30.21063 10409658

[B30] Frohlich H. Boini K. M. Seebohm G. Strutz-Seebohm N. Ureche O. N. Foller M. Eichenmuller M. Shumilina E. Pathare G. Singh A. K. Seidler U. Pfeifer K. E. Lang F. (2011). Hypothyroidism of gene-targeted mice lacking Kcnq1. Pflugers Arch. 461, 45–52 10.1007/s00424-010-0890-5 20978783 PMC3644480

[B31] Gaborit N. Le Bouter S. Szuts V. Varro A. Escande D. Nattel S. Demolombe S. (2007). Regional and tissue specific transcript signatures of ion channel genes in the non-diseased human heart. J. Physiol. (Lond.) 582, 675–693 10.1113/jphysiol.2006.126714 17478540 PMC2075332

[B32] Ghosh S. Silva J. N. Canham R. M. Bowman T. M. Zhang J. Rhee E. K. Woodard P. K. Rudy Y. (2011). Electrophysiologic substrate and intraventricular left ventricular dyssynchrony in nonischemic heart failure patients undergoing cardiac resynchronization therapy. Heart Rhythm 8, 692–699 10.1016/j.hrthm.2011.01.017 21232630 PMC3096066

[B33] Grahammer F. Herling A. W. Lang H. J. Schmitt-Graff A. Wittekindt O. H. Nitschke R. Bleich M. Barhanin J. Warth R. (2001a). The cardiac K+ channel KCNQ1 is essential for gastric acid secretion. Gastroenterology 120, 1363–1371 10.1053/gast.2001.24053 11313306

[B34] Grahammer F. Warth R. Barhanin J. Bleich M. Hug M. J. (2001b). The small conductance K+ channel, KCNQ1: expression, function, and subunit composition in murine trachea. J. Biol. Chem. 276, 42268–42275 10.1074/jbc.M105014200 11527966

[B35] Grunnet M. Jespersen T. Rasmussen H. B. Ljungstrom T. Jorgensen N. K. Olesen S. P. Klaerke D. A. (2002). KCNE4 is an inhibitory subunit to the KCNQ1 channel. J. Physiol. (Lond.) 542, 119–130 10.1113/jphysiol.2002.017301 12096056 PMC2290389

[B36] Haitin Y. Attali B. (2008). The C-terminus of Kv7 channels: a multifunctional module. J. Physiol. (Lond.) 586, 1803–1810 10.1113/jphysiol.2007.149187 18218681 PMC2375714

[B37] Haitin Y. Wiener R. Shaham D. Peretz A. Cohen E. B. Shamgar L. Pongs O. Hirsch J. A. Attali B. (2009). Intracellular domains interactions and gated motions of I(KS) potassium channel subunits. EMBO J. 28, 1994–2005 10.1038/emboj.2009.157 19521339 PMC2718281

[B38] Han W. Bao W. Wang Z. Nattel S. (2002). Comparison of ion-channel subunit expression in canine cardiac Purkinje fibers and ventricular muscle. Circ. Res. 91, 790–797 10.1161/01.RES.0000039534.18114.D9 12411393

[B39] Heitzmann D. Grahammer F. von Hahn T. Schmitt-Graff A. Romeo E. Nitschke R. Gerlach U. Lang H. J. Verrey F. Barhanin J. Warth R. (2004). Heteromeric KCNE2/KCNQ1 potassium channels in the luminal membrane of gastric parietal cells. J. Physiol. (Lond.) 561, 547–557 10.1113/jphysiol.2004.075168 15579540 PMC1665368

[B40] Heitzmann D. Warth R. (2007). No potassium, no acid: K+ channels and gastric acid secretion. Physiology (Bethesda) 22, 335–341 10.1152/physiol.00016.2007 17928547

[B41] Hernandez C. C. Zaika O. Shapiro M. S. (2008). A carboxy-terminal inter-helix linker as the site of phosphatidylinositol 4,5-bisphosphate action on Kv7 (M-type) K+ channels. J. Gen. Physiol. 132, 361–381 10.1085/jgp.200810007 18725531 PMC2518730

[B42] Horikawa N. Suzuki T. Uchiumi T. Minamimura T. Tsukada K. Takeguchi N. Sakai H. (2005). Cyclic AMP-dependent Cl- secretion induced by thromboxane A2 in isolated human colon. J. Physiol. (Lond.) 562, 885–897 10.1113/jphysiol.2004.077776 15611029 PMC1665535

[B43] Hur D. G. Lee J. H. Oh S. H. Kim Y. H. Lee J. H. Shin D. H. Chang S. O. Kim C. S. (2007). KCNQ1/KCNE1 K+ channel and P2Y4 receptor are co-expressed from the time of birth in the apical membrane of rat strial marginal cells. Acta Otolaryngol. Suppl. 558, 30–35 10.1080/03655230701624830 17882567

[B44] Iannotti F. A. Panza E. Barrese V. Viggiano D. Soldovieri M. V. Taglialatela M. (2010). Expression, localization, and pharmacological role of Kv7 potassium channels in skeletal muscle proliferation, differentiation, and survival after myotoxic insults. J. Pharmacol. Exp. Ther. 332, 811–820 10.1124/jpet.109.162800 20040580

[B45] Ikeda S. R. Kammermeier P. J. (2002). M current mystery messenger revealed? Neuron 35, 411–412 10.1016/S0896-6273(02)00792-4 12165463

[B46] Jervell A. Lange-Nielsen F. (1957). Congenital deaf-mutism, functional heart disease with prolongation of the Q-T interval and sudden death. Am. Heart J. 54, 59–68 10.1016/0002-8703(57)90079-0 13435203

[B47] Jespersen T. Membrez M. Nicolas C. S. Pitard B. Staub O. Olesen S. P. Baro I. Abriel H. (2007). The KCNQ1 potassium channel is down-regulated by ubiquitylating enzymes of the Nedd4/Nedd4-like family. Cardiovasc. Res. 74, 64–74 10.1016/j.cardiores.2007.01.008 17289006

[B48] Joshi S. Sedivy V. Hodyc D. Herget J. Gurney A. M. (2009). KCNQ modulators reveal a key role for KCNQ potassium channels in regulating the tone of rat pulmonary artery smooth muscle. J. Pharmacol. Exp. Ther. 329, 368–376 10.1124/jpet.108.147785 19151245 PMC2684066

[B49] Kang C. Tian C. Sonnichsen F. D. Smith J. A. Meiler J. George A. L. Jr. Vanoye C. G. Kim H. J. Sanders C. R. (2008). Structure of KCNE1 and implications for how it modulates the KCNQ1 potassium channel. Biochemistry 47, 7999–8006 10.1021/bi800875q 18611041 PMC2580054

[B50] Kanki H. Kupershmidt S. Yang T. Wells S. Roden D. M. (2004). A structural requirement for processing the cardiac K+ channel KCNQ1. J. Biol. Chem. 279, 33976–33983 10.1074/jbc.M404539200 15140888

[B51] Kim S. J. Greger R. (1999). Voltage-dependent, slowly activating K+ current (I(Ks)) and its augmentation by carbachol in rat pancreatic acini. Pflugers Arch. 438, 604–611 10.1007/s004240051083 10555556

[B52] Knipper M. Claussen C. Ruttiger L. Zimmermann U. Lullmann-Rauch R. Eskelinen E. L. Schroder J. Schwake M. Saftig P. (2006). Deafness in LIMP2-deficient mice due to early loss of the potassium channel KCNQ1/KCNE1 in marginal cells of the stria vascularis. J. Physiol. (Lond.) 576, 73–86 10.1113/jphysiol.2006.116889 16901941 PMC1995639

[B53] Knollmann B. C. Sirenko S. Rong Q. Katchman A. N. Casimiro M. Pfeifer K. Ebert S. N. (2007). Kcnq1 contributes to an adrenergic-sensitive steady-state K+ current in mouse heart. Biochem. Biophys. Res. Commun. 360, 212–218 10.1016/j.bbrc.2007.06.038 17597584 PMC2025686

[B54] Kottgen M. Hoefer A. Kim S. J. Beschorner U. Schreiber R. Hug M. J. Greger R. (1999). Carbachol activates a K+ channel of very small conductance in the basolateral membrane of rat pancreatic acinar cells. Pflugers Arch. 438, 597–603 10.1007/s004240051082 10555555

[B55] Kunzelmann K. Hubner M. Schreiber R. Levy-Holzman R. Garty H. Bleich M. Warth R. Slavik M. von Hahn T. Greger R. (2001). Cloning and function of the rat colonic epithelial K+ channel KVLQT1. J. Membr. Biol. 179, 155–164 10.1007/s002320010045 11220365

[B56] Kurokawa J. Motoike H. K. Kass R. S. (2001). TEA(+)-sensitive KCNQ1 constructs reveal pore-independent access to KCNE1 in assembled I(Ks) channels. J. Gen. Physiol. 117, 43–52 10.1085/jgp.117.1.43 11134230 PMC2232469

[B57] Kurokawa J. Motoike H. K. Rao J. Kass R. S. (2004). Regulatory actions of the A-kinase anchoring protein Yotiao on a heart potassium channel downstream of PKA phosphorylation. Proc. Natl. Acad. Sci. U.S.A. 101, 16374–16378 10.1073/pnas.0405583101 15528278 PMC525330

[B58] Lambrecht N. W. Yakubov I. Scott D. Sachs G. (2005). Identification of the K efflux channel coupled to the gastric H-K-ATPase during acid secretion. Physiol. Genomics 21, 81–91 10.1152/physiolgenomics.00212.2004 15613615

[B59] Lee J. E. Park H. S. Uhm D. Y. Kim S. J. (2004). Effects of KCNQ1 channel blocker, 293B, on the acetylcholine-induced Cl- secretion of rat pancreatic acini. Pancreas 28, 435–442 10.1097/00006676-200405000-00013 15097862

[B60] Lee M. P. Ravenel J. D. Hu R. J. Lustig L. R. Tomaselli G. Berger R. D. Brandenburg S. A. Litzi T. J. Bunton T. E. Limb C. Francis H. Gorelikow M. Gu H. Washington K. Argani P. Goldenring J. R. Coffey R. J. Feinberg A. P. (2000). Targeted disruption of the Kvlqt1 gene causes deafness and gastric hyperplasia in mice. J. Clin. Invest. 106, 1447–1455 10.1172/JCI10897 11120752 PMC387258

[B61] Lee W. K. Torchalski B. Roussa E. Thevenod F. (2008). Evidence for KCNQ1 K+ channel expression in rat zymogen granule membranes and involvement in cholecystokinin-induced pancreatic acinar secretion. Am. J. Physiol. Cell Physiol. 294, C879–C892 10.1152/ajpcell.00403.2007 18216164

[B62] Lerche C. Bruhova I. Lerche H. Steinmeyer K. Wei A. D. Strutz-Seebohm N. Lang F. Busch A. E. Zhorov B. S. Seebohm G. (2007). Chromanol 293B binding in KCNQ1 (Kv7.1) channels involves electrostatic interactions with a potassium ion in the selectivity filter. Mol. Pharmacol. 71, 1503–1511 10.1124/mol.106.031682 17347319

[B63] Lerche C. Seebohm G. Wagner C. I. Scherer C. R. Dehmelt L. Abitbol I. Gerlach U. Brendel J. Attali B. Busch A. E. (2000). Molecular impact of MinK on the enantiospecific block of I(Ks) by chromanols. Br. J. Pharmacol. 131, 1503–1506 10.1038/sj.bjp.0703734 11139424 PMC1572493

[B64] Li Y. Zaydman M. A. Wu D. Shi J. Guan M. Virgin-Downey B. Cui J. (2011). KCNE1 enhances phosphatidylinositol 4,5-bisphosphate (PIP2) sensitivity of IKs to modulate channel activity. Proc. Natl. Acad. Sci. U.S.A. 108, 9095–9100 10.1073/pnas.1109773109 21576493 PMC3107281

[B65] Liang G. H. Jin Z. Ulfendahl M. Jarlebark L. (2006). Molecular analyses of KCNQ1-5 potassium channel mRNAs in rat and guinea pig inner ears: expression, cloning, and alternative splicing. Acta Otolaryngol. 126, 346–352 10.1080/00016480500416777 16608784

[B66] Loussouarn G. Park K. H. Bellocq C. Baro I. Charpentier F. Escande D. (2003). Phosphatidylinositol-4,5-bisphosphate, PIP2, controls KCNQ1/KCNE1 voltage-gated potassium channels: a functional homology between voltage-gated and inward rectifier K+ channels. EMBO J. 22, 5412–5421 10.1093/emboj/cdg526 14532114 PMC213780

[B67] Lvov A. Gage S. D. Berrios V. M. Kobertz W. R. (2010). Identification of a protein-protein interaction between KCNE1 and the activation gate machinery of KCNQ1. J. Gen. Physiol. 135, 607–618 10.1085/jgp.200910386 20479109 PMC2888057

[B68] Ma L. J. Ohmert I. Vardanyan V. (2011). Allosteric features of KCNQ1 gating revealed by alanine scanning mutagenesis. Biophys. J. 100, 885–894 10.1016/j.bpj.2010.12.2825 21320432 PMC3037572

[B69] Macedo-Ribeiro S. Hemrika W. Renirie R. Wever R. Messerschmidt A. (1999). X-ray crystal structures of active site mutants of the vanadium-containing chloroperoxidase from the fungus *Curvularia inaequalis*. J. Biol. Inorg. Chem. 4, 209–219 10.1007/s007750050306 10499093

[B70] Mackie A. R. Brueggemann L. I. Henderson K. K. Shiels A. J. Cribbs L. L. Scrogin K. E. Byron K. L. (2008). Vascular KCNQ potassium channels as novel targets for the control of mesenteric artery constriction by vasopressin, based on studies in single cells, pressurized arteries, and in vivo measurements of mesenteric vascular resistance. J. Pharmacol. Exp. Ther. 325, 475–483 10.1124/jpet.107.135764 18272810 PMC2597077

[B71] Mall M. Wissner A. Schreiber R. Kuehr J. Seydewitz H. H. Brandis M. Greger R. Kunzelmann K. (2000). Role of K(V)LQT1 in cyclic adenosine monophosphate-mediated Cl(-) secretion in human airway epithelia. Am. J. Respir. Cell Mol. Biol. 23, 283–289 10970817 10.1165/ajrcmb.23.3.4060

[B72] Marcus D. C. Shen Z. (1994). Slowly activating voltage-dependent K+ conductance is apical pathway for K+ secretion in vestibular dark cells. Am. J. Physiol. 267, C857–C864 7943212 10.1152/ajpcell.1994.267.3.C857

[B73] Marx S. O. Kurokawa J. Reiken S. Motoike H. D’Armiento J. Marks A. R. Kass R. S. (2002). Requirement of a macromolecular signaling complex for beta adrenergic receptor modulation of the KCNQ1-KCNE1 potassium channel. Science 295, 496–499 10.1126/science.1066843 11799244

[B74] Mazhari R. Nuss H. B. Armoundas A. A. Winslow R. L. Marban E. (2002). Ectopic expression of KCNE3 accelerates cardiac repolarization and abbreviates the QT interval. J. Clin. Invest. 109, 1083–1090 10.1172/JCI15062 11956246 PMC150950

[B75] McCallum L. A. Greenwood I. A. Tribe R. M. (2009). Expression and function of K(v)7 channels in murine myometrium throughout oestrous cycle. Pflugers Arch. 457, 1111–1120 10.1007/s00424-008-0567-5 18709386

[B76] McCrossan Z. A. Abbott G. W. (2004). The MinK-related peptides. Neuropharmacology 47, 787–821 10.1016/j.neuropharm.2004.06.018 15527815

[B77] Melman Y. F. Domenech A. de la Luna S. McDonald T. V. (2001). Structural determinants of KvLQT1 control by the KCNE family of proteins. J. Biol. Chem. 276, 6439–6444 10.1074/jbc.M103717200 11104781

[B78] Melman Y. F. Krumerman A. McDonald T. V. (2002). A single transmembrane site in the KCNE-encoded proteins controls the specificity of KvLQT1 channel gating. J. Biol. Chem. 277, 25187–25194 10.1074/jbc.M200564200 11994278

[B79] Melman Y. F. Um S. Y. Krumerman A. Kagan A. McDonald T. V. (2004). KCNE1 binds to the KCNQ1 pore to regulate potassium channel activity. Neuron 42, 927–937 10.1016/j.neuron.2004.06.001 15207237

[B80] Morin T. J. Kobertz W. R. (2008). Counting membrane-embedded KCNE beta-subunits in functioning K+ channel complexes. Proc. Natl. Acad. Sci. U.S.A. 105, 1478–1482 10.1073/pnas.0710366105 18223154 PMC2234169

[B81] Morokuma J. Blackiston D. Adams D. S. Seebohm G. Trimmer B. Levin M. (2008). Modulation of potassium channel function confers a hyperproliferative invasive phenotype on embryonic stem cells. Proc. Natl. Acad. Sci. U.S.A. 105, 16608–16613 10.1073/pnas.0808328105 18931301 PMC2575467

[B82] Nakajo K. Kubo Y. (2007). KCNE1 and KCNE3 stabilize and/or slow voltage sensing S4 segment of KCNQ1 channel. J. Gen. Physiol. 130, 269–281 10.1085/jgp.200709805 17698596 PMC2151641

[B83] Nakajo K. Kubo Y. (2008). Second coiled-coil domain of KCNQ channel controls current expression and subfamily specific heteromultimerization by salt bridge networks. J. Physiol. (Lond.) 586, 2827–2840 10.1113/jphysiol.2007.148601 18440995 PMC2517212

[B84] Nakajo K. Nishino A. Okamura Y. Kubo Y. (2011). KCNQ1 subdomains involved in KCNE modulation revealed by an invertebrate KCNQ1 orthologue. J. Gen. Physiol. 138, 521–535 10.1085/jgp.201110677 22042987 PMC3206303

[B85] Nakajo K. Ulbrich M. H. Kubo Y. Isacoff E. Y. (2010). Stoichiometry of the KCNQ1 – KCNE1 ion channel complex. Proc. Natl. Acad. Sci. U.S.A. 107, 18862–18867 10.1073/pnas.1010354107 20962273 PMC2973890

[B86] Neyroud N. Tesson F. Denjoy I. Leibovici M. Donger C. Barhanin J. Faure S. Gary F. Coumel P. Petit C. Schwartz K. Guicheney P. (1997). A novel mutation in the potassium channel gene KVLQT1 causes the Jervell and Lange-Nielsen cardioauditory syndrome. Nat. Genet. 15, 186–189 10.1038/nbt0297-186 9020846

[B87] Ng F. L. Davis A. J. Jepps T. A. Harhun M. I. Yeung S. Y. Wan A. Reddy M. Melville D. Nardi A. Khong T. K. Greenwood I. A. (2011). Expression and function of the K+ channel KCNQ genes in human arteries. Br. J. Pharmacol. 162, 42–53 10.1111/j.1476-5381.2010.01027.x 20840535 PMC3012405

[B88] Nicolas C. S. Park K. H. El Harchi A. Camonis J. Kass R. S. Escande D. Merot J. Loussouarn G. Le Bouffant F. Baro I. (2008). IKs response to protein kinase A-dependent KCNQ1 phosphorylation requires direct interaction with microtubules. Cardiovasc. Res. 79, 427–435 10.1093/cvr/cvn085 18390900 PMC2781743

[B89] Nicolas M. Dememes D. Martin A. Kupershmidt S. Barhanin J. (2001). KCNQ1/KCNE1 potassium channels in mammalian vestibular dark cells. Hear. Res. 153, 132–145 10.1016/S0378-5955(00)00268-9 11223304

[B90] Ohya S. Sergeant G. P. Greenwood I. A. Horowitz B. (2003). Molecular variants of KCNQ channels expressed in murine portal vein myocytes: a role in delayed rectifier current. Circ. Res. 92, 1016–1023 10.1161/01.RES.0000070880.20955.F4 12690036

[B91] Osteen J. D. Gonzalez C. Sampson K. J. Iyer V. Rebolledo S. Larsson H. P. Kass R. S. (2010). KCNE1 alters the voltage sensor movements necessary to open the KCNQ1 channel gate. Proc. Natl. Acad. Sci. U.S.A. 107, 22710–22715 10.1073/pnas.1014150107 21149716 PMC3012494

[B92] Panaghie G. Tai K. K. Abbott G. W. (2006). Interaction of KCNE subunits with the KCNQ1 K+ channel pore. J. Physiol. (Lond.) 570, 455–467 10.1113/jphysiol.2005.100644 16308347 PMC1479883

[B93] Park K. S. Pang B. Park S. J. Lee Y. G. Bae J. Y. Park S. Kim I. Kim S. J. (2011). Identification and functional characterization of ion channels in CD34(+) hematopoietic stem cells from human peripheral blood. Mol. Cells 32, 181–188 10.1007/s10059-011-0122-7 21638203 PMC3887668

[B94] Piccini M. Vitelli F. Seri M. Galietta L. J. Moran O. Bulfone A. Banfi S. Pober B. Renieri A. (1999). KCNE1-like gene is deleted in AMME contiguous gene syndrome: identification and characterization of the human and mouse homologs. Genomics 60, 251–257 10.1006/geno.1999.5904 10493825

[B95] Pugsley M. K. Towart R. Authier S. Gallacher D. J. Curtis M. J. (2011). Innovation in safety pharmacology testing. J. Pharmacol. Toxicol. Methods 64, 1–6 10.1016/j.vascn.2011.03.008 21640842

[B96] Pusch M. (1998). Increase of the single-channel conductance of KvLQT1 potassium channels induced by the association with minK. Pflugers Arch. 437, 172–174 10.1007/s004240050765 9817805

[B97] Pusch M. Magrassi R. Wollnik B. Conti F. (1998). Activation and inactivation of homomeric KvLQT1 potassium channels. Biophys. J. 75, 785–792 10.1016/S0006-3495(98)77568-X 9675180 PMC1299753

[B98] Rasmussen H. B. Moller M. Knaus H. G. Jensen B. S. Olesen S. P. Jorgensen N. K. (2004). Subcellular localization of the delayed rectifier K(+) channels KCNQ1 and ERG1 in the rat heart. Am. J. Physiol. Heart Circ. Physiol. 286, H1300–H1309 10.1152/ajpheart.00344.2003 14670813

[B99] Rocheleau J. M. Kobertz W. R. (2008). KCNE peptides differently affect voltage sensor equilibrium and equilibration rates in KCNQ1 K+ channels. J. Gen. Physiol. 131, 59–68 10.1085/jgp.200709816 18079560 PMC2174159

[B100] Romey G. Attali B. Chouabe C. Abitbol I. Guillemare E. Barhanin J. Lazdunski M. (1997). Molecular mechanism and functional significance of the MinK control of the KvLQT1 channel activity. J. Biol. Chem. 272, 16713–16716 10.1074/jbc.272.27.16713 9201970

[B101] Roura-Ferrer M. Sole L. Martinez-Marmol R. Villalonga N. Felipe A. (2008). Skeletal muscle Kv7 (KCNQ) channels in myoblast differentiation and proliferation. Biochem. Biophys. Res. Commun. 369, 1094–1097 10.1016/j.bbrc.2008.02.152 18331828

[B102] Sanguinetti M. C. Curran M. E. Zou A. Shen J. Spector P. S. Atkinson D. L. Keating M. T. (1996). Coassembly of K(V)LQT1 and minK (IsK) proteins to form cardiac I(Ks) potassium channel. Nature 384, 80–83 10.1038/384080a0 8900283

[B103] Sanguinetti M. C. Jurkiewicz N. K. (1990). Two components of cardiac delayed rectifier K+ current. Differential sensitivity to block by class III antiarrhythmic agents. J. Gen. Physiol. 96, 195–215 10.1085/jgp.96.1.195 2170562 PMC2228985

[B104] Schmitt N. Schwarz M. Peretz A. Abitbol I. Attali B. Pongs O. (2000). A recessive C-terminal Jervell and Lange-Nielsen mutation of the KCNQ1 channel impairs subunit assembly. EMBO J. 19, 332–340 10.1093/emboj/19.3.332 10654932 PMC305570

[B105] Schroeder B. C. Waldegger S. Fehr S. Bleich M. Warth R. Greger R. Jentsch T. J. (2000). A constitutively open potassium channel formed by KCNQ1 and KCNE3. Nature 403, 196–199 10.1038/35003200 10646604

[B106] Schulze-Bahr E. Wang Q. Wedekind H. Haverkamp W. Chen Q. Sun Y. Rubie C. Hordt M. Towbin J. A. Borggrefe M. Assmann G. Qu X. Somberg J. C. Breithardt G. Oberti C. Funke H. (1997). KCNE1 mutations cause Jervell and Lange-Nielsen syndrome. Nat. Genet. 17, 267–268 10.1038/ng1197-267 9354783

[B107] Schwake M. Jentsch T. J. Friedrich T. (2003). A carboxy-terminal domain determines the subunit specificity of KCNQ K+ channel assembly. EMBO Rep. 4, 76–81 10.1038/sj.embor.embor715 12524525 PMC1315815

[B108] Seebohm G. Chen J. Strutz N. Culberson C. Lerche C. Sanguinetti M. C. (2003a). Molecular determinants of KCNQ1 channel block by a benzodiazepine. Mol. Pharmacol. 64, 70–77 10.1124/mol.64.1.70 12815162

[B109] Seebohm G. Pusch M. Chen J. Sanguinetti M. C. (2003b). Pharmacological activation of normal and arrhythmia-associated mutant KCNQ1 potassium channels. Circ. Res. 93, 941–947 10.1161/01.RES.0000102866.67863.2B 14576198

[B110] Seebohm G. Sanguinetti M. C. Pusch M. (2003c). Tight coupling of rubidium conductance and inactivation in human KCNQ1 potassium channels. J. Physiol. (Lond.) 552, 369–378 10.1113/jphysiol.2003.046490 14561821 PMC2343369

[B111] Seebohm G. Lerche C. Busch A. E. Bachmann A. (2001a). Dependence of I(Ks) biophysical properties on the expression system. Pflugers Arch. 442, 891–895 10.1007/s004240100608 11680622

[B112] Seebohm G. Lerche C. Pusch M. Steinmeyer K. Bruggemann A. Busch A. E. (2001b). A kinetic study on the stereospecific inhibition of KCNQ1 and I(Ks) by the chromanol 293B. Br. J. Pharmacol. 134, 1647–1654 10.1038/sj.bjp.0704421 11739240 PMC1572901

[B113] Seebohm G. Scherer C. R. Busch A. E. Lerche C. (2001c). Identification of specific pore residues mediating KCNQ1 inactivation. A novel mechanism for long QT syndrome. J. Biol. Chem. 276, 13600–13605 11278406 10.1074/jbc.M008373200

[B114] Seebohm G. Strutz-Seebohm N. Ureche O. N. Baltaev R. Lampert A. Kornichuk G. Kamiya K. Wuttke T. V. Lerche H. Sanguinetti M. C. Lang F. (2006). Differential roles of S6 domain hinges in the gating of KCNQ potassium channels. Biophys. J. 90, 2235–2244 10.1529/biophysj.105.067165 16326905 PMC1386802

[B115] Seebohm G. Westenskow P. Lang F. Sanguinetti M. C. (2005). Mutation of colocalized residues of the pore helix and transmembrane segments S5 and S6 disrupt deactivation and modify inactivation of KCNQ1 K+ channels. J. Physiol. (Lond.) 563, 359–368 10.1113/jphysiol.2004.080887 15649981 PMC1665586

[B116] Sesti F. Goldstein S. A. (1998). Single-channel characteristics of wild-type IKs channels and channels formed with two minK mutants that cause long QT syndrome. J. Gen. Physiol. 112, 651–663 10.1085/jgp.112.6.651 9834138 PMC2229448

[B117] Shamgar L. Haitin Y. Yisharel I. Malka E. Schottelndreier H. Peretz A. Paas Y. Attali B. (2008). KCNE1 constrains the voltage sensor of Kv7.1 K+ channels. PLoS ONE 3, e1943 10.1371/journal.pone.0001943 18398469 PMC2275793

[B118] Shen Z. Liu J. Marcus D. C. Shiga N. Wangemann P. (1995). DIDS increases K+ secretion through an IsK channel in apical membrane of vestibular dark cell epithelium of gerbil. J. Membr. Biol. 146, 283–291 8568843 10.1007/BF00233948

[B119] Silva J. Rudy Y. (2005). Subunit interaction determines IKs participation in cardiac repolarization and repolarization reserve. Circulation 112, 1384–1391 10.1161/CIRCULATIONAHA.105.543306 16129795 PMC1820744

[B120] Silva J. R. Pan H. Wu D. Nekouzadeh A. Decker K. F. Cui J. Baker N. A. Sept D. Rudy Y. (2009). A multiscale model linking ion-channel molecular dynamics and electrostatics to the cardiac action potential. Proc. Natl. Acad. Sci. U.S.A. 106, 11102–11106 10.1073/pnas.0904505106 19549851 PMC2700153

[B121] Silva J. R. Rudy Y. (2010). Multi-scale electrophysiology modeling: from atom to organ. J. Gen. Physiol. 135, 575–581 10.1085/jgp.200910358 20513759 PMC2888060

[B122] Smith J. A. Vanoye C. G. George A. L. Jr. Meiler J. Sanders C. R. (2007). Structural models for the KCNQ1 voltage-gated potassium channel. Biochemistry 46, 14141–14152 10.1021/bi700637a 17999538 PMC2565492

[B123] Strutz-Seebohm N. Pusch M. Wolf S. Stoll R. Tapken D. Gerwert K. Attali B. Seebohm G. (2011). Structural basis of slow activation gating in the cardiac I Ks channel complex. Cell. Physiol. Biochem. 27, 443–452 10.1159/000329965 21691061

[B124] Strutz-Seebohm N. Seebohm G. Fedorenko O. Baltaev R. Engel J. Knirsch M. Lang F. (2006). Functional coassembly of KCNQ4 with KCNE-beta- subunits in Xenopus oocytes. Cell. Physiol. Biochem. 18, 57–66 10.1159/000097675 16914890

[B125] Sugimoto T. Tanabe Y. Shigemoto R. Iwai M. Takumi T. Ohkubo H. Nakanishi S. (1990). Immunohistochemical study of a rat membrane protein which induces a selective potassium permeation: its localization in the apical membrane portion of epithelial cells. J. Membr. Biol. 113, 39–47 10.1007/BF01869604 2154581

[B126] Sunose H. Liu J. Shen Z. Marcus D. C. (1997). cAMP increases apical IsK channel current and K+ secretion in vestibular dark cells. J. Membr. Biol. 156, 25–35 10.1007/s002329900184 9070461

[B127] Tai K. K. Goldstein S. A. (1998). The conduction pore of a cardiac potassium channel. Nature 391, 605–608 10.1038/35416 9468141

[B128] Takumi T. Moriyoshi K. Aramori I. Ishii T. Oiki S. Okada Y. Ohkubo H. Nakanishi S. (1991). Alteration of channel activities and gating by mutations of slow ISK potassium channel. J. Biol. Chem. 266, 22192–22198 1939241

[B129] Takumi T. Ohkubo H. Nakanishi S. (1988). Cloning of a membrane protein that induces a slow voltage-gated potassium current. Science 242, 1042–1045 10.1126/science.3194754 3194754

[B130] Tapper A. R. George A. L. Jr. (2000). MinK subdomains that mediate modulation of and association with KvLQT1. J. Gen. Physiol. 116, 379–390 10.1085/jgp.116.3.379 10962015 PMC2233688

[B131] Tapper A. R. George A. L. Jr. (2001). Location and orientation of minK within the I(Ks) potassium channel complex. J. Biol. Chem. 276, 38249–38254 11479291 10.1074/jbc.M103956200

[B132] Thomas A. M. Harmer S. C. Khambra T. Tinker A. (2011). Characterization of a binding site for anionic phospholipids on KCNQ1. J. Biol. Chem. 286, 2088–2100 10.1074/jbc.M110.153551 21084310 PMC3023506

[B133] Tian C. Vanoye C. G. Kang C. Welch R. C. Kim H. J. George A. L. Jr. Sanders C. R. (2007). Preparation, functional characterization, and NMR studies of human KCNE1, a voltage-gated potassium channel accessory subunit associated with deafness and long QT syndrome. Biochemistry 46, 11459–11472 10.1021/bi700705j 17892302 PMC2565491

[B134] Tinel N. Diochot S. Borsotto M. Lazdunski M. Barhanin J. (2000). KCNE2 confers background current characteristics to the cardiac KCNQ1 potassium channel. EMBO J. 19, 6326–6330 10.1093/emboj/19.23.6326 11101505 PMC305874

[B135] Towart R. Linders J. T. Hermans A. N. Rohrbacher J. van der Linde H. J. Ercken M. Cik M. Roevens P. Teisman A. Gallacher D. J. (2009). Blockade of the I(Ks) potassium channel: an overlooked cardiovascular liability in drug safety screening? J. Pharmacol. Toxicol. Methods 60, 1–10 10.1016/j.vascn.2009.04.197 19439185

[B136] Tristani-Firouzi M. Sanguinetti M. C. (1998). Voltage-dependent inactivation of the human K+ channel KvLQT1 is eliminated by association with minimal K+ channel (minK) subunits. J. Physiol. (Lond.) 510(Pt 1), 37–45 10.1111/j.1469-7793.1998.037bz.x 9625865 PMC2231024

[B137] Tzounopoulos T. Maylie J. Adelman J. P. (1998). Gating of I(sK) channels expressed in Xenopus oocytes. Biophys. J. 74, 2299–2305 10.1016/S0006-3495(98)77939-1 9591657 PMC1299573

[B138] Vallon V. Grahammer F. Richter K. Bleich M. Lang F. Barhanin J. Volkl H. Warth R. (2001). Role of KCNE1-dependent K+ fluxes in mouse proximal tubule. J. Am. Soc. Nephrol. 12, 2003–2011 11562398 10.1681/ASN.V12102003

[B139] Vallon V. Grahammer F. Volkl H. Sandu C. D. Richter K. Rexhepaj R. Gerlach U. Rong Q. Pfeifer K. Lang F. (2005). KCNQ1-dependent transport in renal and gastrointestinal epithelia. Proc. Natl. Acad. Sci. U.S.A. 102, 17864–17869 10.1073/pnas.0505860102 16314573 PMC1308898

[B140] Wang K. Terrenoire C. Sampson K. J. Iyer V. Osteen J. D. Lu J. Keller G. Kotton D. N. Kass R. S. (2011a). Biophysical properties of slow potassium channels in human embryonic stem cell derived cardiomyocytes implicate subunit stoichiometry. J. Physiol. (Lond.) 589, 6093–6104 10.1113/jphysiol.2010.203463 22025662 PMC3286688

[B141] Wang Y. H. Jiang M. Xu X. L. Hsu K. L. Zhang M. Tseng G. N. (2011b). Gating-related molecular motions in the extracellular domain of the IKs channel: implications for IKs channelopathy. J. Membr. Biol. 239, 137–156 10.1007/s00232-010-9333-7 21152909 PMC3893887

[B142] Wang K. W. Goldstein S. A. (1995). Subunit composition of minK potassium channels. Neuron 14, 1303–1309 10.1016/0896-6273(95)90277-5 7605639

[B143] Wang K. W. Tai K. K. Goldstein S. A. (1996a). MinK residues line a potassium channel pore. Neuron 16, 571–577 10.1016/S0896-6273(00)80076-8 8785054

[B144] Wang Q. Curran M. E. Splawski I. Burn T. C. Millholland J. M. VanRaay T. J. Shen J. Timothy K. W. Vincent G. M. de Jager T. Schwartz P. J. Toubin J. A. Moss A. J. Atkinson D. L. Landes G. M. Connors T. D. Keating M. T. (1996b). Positional cloning of a novel potassium channel gene: KVLQT1 mutations cause cardiac arrhythmias. Nat. Genet. 12, 17–23 10.1038/ng0196-17 8528244

[B145] Wang W. Xia J. Kass R. S. (1998). MinK-KvLQT1 fusion proteins, evidence for multiple stoichiometries of the assembled IsK channel. J. Biol. Chem. 273, 34069–34074 10.1074/jbc.273.38.24867 9852064

[B146] Wangemann P. (1995). Comparison of ion transport mechanisms between vestibular dark cells and strial marginal cells. Hear. Res. 90, 149–157 10.1016/0378-5955(95)00157-2 8974992

[B147] Wangemann P. Liu J. Marcus D. C. (1995). Ion transport mechanisms responsible for K+ secretion and the transepithelial voltage across marginal cells of stria vascularis in vitro. Hear. Res. 84, 19–29 10.1016/0378-5955(95)00009-S 7642451

[B148] Warth R. Barhanin J. (2002). The multifaceted phenotype of the knockout mouse for the KCNE1 potassium channel gene. Am. J. Physiol. Regul. Integr. Comp. Physiol. 282, R639–R648 11832382 10.1152/ajpregu.00649.2001

[B149] Wu D. M. Jiang M. Zhang M. Liu X. S. Korolkova Y. V. Tseng G. N. (2006). KCNE2 is colocalized with KCNQ1 and KCNE1 in cardiac myocytes and may function as a negative modulator of I(Ks) current amplitude in the heart. Heart Rhythm 3, 1469–1480 10.1016/j.hrthm.2006.08.019 17161791

[B150] Xu Q. Minor D. L. Jr. (2009). Crystal structure of a trimeric form of the K(V)7.1 (KCNQ1) A-domain tail coiled-coil reveals structural plasticity and context dependent changes in a putative coiled-coil trimerization motif. Protein Sci. 18, 2100–2114 10.1002/pro.105 19693805 PMC2786974

[B151] Xu X. Jiang M. Hsu K. L. Zhang M. Tseng G. N. (2008). KCNQ1 and KCNE1 in the IKs channel complex make state-dependent contacts in their extracellular domains. J. Gen. Physiol. 131, 589–603 10.1085/jgp.200809976 18504315 PMC2391252

[B152] Yang W. P. Levesque P. C. Little W. A. Conder M. L. Shalaby F. Y. Blanar M. A. (1997). KvLQT1, a voltage-gated potassium channel responsible for human cardiac arrhythmias. Proc. Natl. Acad. Sci. U.S.A. 94, 4017–4021 10.1073/pnas.94.26.14524 9108097 PMC20560

[B153] Yeung S. Y. Pucovsky V. Moffatt J. D. Saldanha L. Schwake M. Ohya S. Greenwood I. A. (2007). Molecular expression and pharmacological identification of a role for K(v)7 channels in murine vascular reactivity. Br. J. Pharmacol. 151, 758–770 10.1038/sj.bjp.0707284 17519950 PMC2014117

[B154] Zhang H. Craciun L. C. Mirshahi T. Rohacs T. Lopes C. M. Jin T. Logothetis D. E. (2003). PIP(2) activates KCNQ channels, and its hydrolysis underlies receptor-mediated inhibition of M currents. Neuron 37, 963–975 10.1016/S0896-6273(03)00125-9 12670425

[B155] Zheng R. Thompson K. Obeng-Gyimah E. Alessi D. Chen J. Cheng H. McDonald T. V. (2010). Analysis of the interactions between the C-terminal cytoplasmic domains of KCNQ1 and KCNE1 channel subunits. Biochem. J. 428, 75–84 10.1042/BJ20090977 20196769 PMC2888147

[B156] Zhong X. Z. Harhun M. I. Olesen S. P. Ohya S. Moffatt J. D. Cole W. C. Greenwood I. A. (2010). Participation of KCNQ (Kv7) potassium channels in myogenic control of cerebral arterial diameter. J. Physiol. (Lond.) 588, 3277–3293 10.1113/jphysiol.2010.192823 20624791 PMC2976022

[B157] Zicha S. Moss I. Allen B. Varro A. Papp J. Dumaine R. Antzelevich C. Nattel S. (2003). Molecular basis of species-specific expression of repolarizing K+ currents in the heart. Am. J. Physiol. Heart Circ. Physiol. 285, H1641–H1649 12816752 10.1152/ajpheart.00346.2003

